# Advanced Health Information Technologies to Engage Parents, Clinicians, and Community Nutritionists in Coordinating Responsive Parenting Care: Descriptive Case Series of the Women, Infants, and Children Enhancements to Early Healthy Lifestyles for Baby (WEE Baby) Care Randomized Controlled Trial

**DOI:** 10.2196/22121

**Published:** 2020-11-24

**Authors:** Samantha MR Kling, Holly A Harris, Michele Marini, Adam Cook, Lindsey B Hess, Shawnee Lutcher, Jacob Mowery, Scott Bell, Sandra Hassink, Shannon B Hayward, Greg Johnson, Jennifer Franceschelli Hosterman, Ian M Paul, Christopher Seiler, Shirley Sword, Jennifer S Savage, Lisa Bailey-Davis

**Affiliations:** 1 Center for Childhood Obesity Research The Pennsylvania State University University Park, PA United States; 2 Evaluation Sciences Unit, Division of Primary Care and Population Health Department of Medicine, School of Medicine Stanford University Stanford, CA United States; 3 Department of Nutritional Sciences The Pennsylvania State University University Park, PA United States; 4 Geisinger Obesity Institute Geisinger Danville, PA United States; 5 Erasmus Medical Center Generation R Study University Medical Center Rotterdam Rotterdam Netherlands; 6 Bureau of Women, Infants, and Children Pennsylvania Department of Health Harrisburg, PA United States; 7 Institute for Healthy Childhood Weight American Academy of Pediatrics Wilmington, DE United States; 8 Maternal and Family Health Services Wilkes-Barre, PA United States; 9 Department of Pediatrics Penn State College of Medicine Hershey, PA United States; 10 Department of Public Health Sciences Penn State College of Medicine Hershey, PA United States; 11 Department of Population Health Sciences Geisinger Danville, PA United States

**Keywords:** early obesity prevention, responsive parenting, health information technology, coordination of care, clinical care, pragmatic intervention, data sharing

## Abstract

**Background:**

Socioeconomically disadvantaged newborns receive care from primary care providers (PCPs) and Women, Infants, and Children (WIC) nutritionists. However, care is not coordinated between these settings, which can result in conflicting messages. Stakeholders support an integrated approach that coordinates services between settings with care tailored to patient-centered needs.

**Objective:**

This analysis describes the usability of advanced health information technologies aiming to engage parents in self-reporting parenting practices, integrate data into electronic health records to inform and facilitate documentation of provided responsive parenting (RP) care, and share data between settings to create opportunities to coordinate care between PCPs and WIC nutritionists.

**Methods:**

Parents and newborns (dyads) who were eligible for WIC care and received pediatric care in a single health system were recruited and randomized to a RP intervention or control group. For the 6-month intervention, electronic systems were created to facilitate documentation, data sharing, and coordination of provided RP care. Prior to PCP visits, parents were prompted to respond to the Early Healthy Lifestyles (EHL) self-assessment tool to capture current RP practices. Responses were integrated into the electronic health record and shared with WIC. Documentation of RP care and an 80-character, free-text comment were shared between WIC and PCPs. A care coordination opportunity existed when the dyad attended a WIC visit and these data were available from the PCP, and vice versa. Care coordination was demonstrated when WIC or PCPs interacted with data and documented RP care provided at the visit.

**Results:**

Dyads (N=131) attended 459 PCP (3.5, SD 1.0 per dyad) and 296 WIC (2.3, SD 1.0 per dyad) visits. Parents completed the EHL tool prior to 53.2% (244/459) of PCP visits (1.9, SD 1.2 per dyad), PCPs documented provided RP care at 35.3% (162/459) of visits, and data were shared with WIC following 100% (459/459) of PCP visits. A WIC visit followed a PCP visit 50.3% (231/459) of the time; thus, there were 1.8 (SD 0.8 per dyad) PCP to WIC care coordination opportunities. WIC coordinated care by documenting RP care at 66.7% (154/231) of opportunities (1.2, SD 0.9 per dyad). WIC visits were followed by a PCP visit 58.9% (116/197) of the time; thus, there were 0.9 (SD 0.8 per dyad) WIC to PCP care coordination opportunities. PCPs coordinated care by documenting RP care at 44.0% (51/116) of opportunities (0.4, SD 0.6 per dyad).

**Conclusions:**

Results support the usability of advanced health information technology strategies to collect patient-reported data and share these data between multiple providers. Although PCPs and WIC shared data, WIC nutritionists were more likely to use data and document RP care to coordinate care than PCPs. Variability in timing, sequence, and frequency of visits underscores the need for flexibility in pragmatic studies.

**Trial Registration:**

ClinicalTrials.gov NCT03482908; https://clinicaltrials.gov/ct2/show/NCT03482908

**International Registered Report Identifier (IRRID):**

RR2-10.1186/s12887-018-1263-z

## Introduction

Most lower-income children in the United States receive frequent preventive care, with up to 7 visits in the first 6 months after birth from primary care providers (PCPs) and the Special Supplemental Nutrition Program for Women, Infants, and Children (WIC) [1-3]. Fragmentation across care settings can lead to inconsistent prevention messages and caregiver confusion [4]. A solution to coordinate care among parents, clinicians, and community health professionals is needed [4]. Stakeholders are aligned in their support of a solution that both personalizes messaging to meet parents’ time-sensitive needs on infant development and integrates relevant data to coordinate care across settings and break down silos [4]. The perceived benefits of personalization, integration, and care coordination, according to stakeholders, include improved child health outcomes and reduced duplication of efforts [4].

From a preventive health model perspective, the need to collect and share data among patients and clinical and community health professionals aligns with a series of reports [5-9] calling for systems approaches through the integration and coordination of care across service settings [9,10]. Integrated and coordinated care approaches are commonly applied within the clinical sector [11-13] (eg, general physicians and specialists), but given the capabilities and demonstrated benefits of advanced health information technologies (HITs) [12,13], the contemporary calls aim to extend these approaches to public health, community settings, and patients. In particular, the Chronic Care Model and the Culture of Health Action Framework emphasize connectivity and integration of public health, clinical services, and social services to advance quality, health outcomes, and equity, specifically for vulnerable populations [14,15].

The integrated and coordinated care model described in this paper applied advanced HIT strategies and represents a novel cross-sector care delivery model to address patient-centered care [11,16]. As little is known about the feasibility of cross-sector delivery models, this study addresses a gap by applying and evaluating the usability of advanced HIT capabilities to engage patients and clinical and community providers in a broader patient-centered, integrated, and coordinated care process. The intervention arm of the WIC Enhancements to Early Healthy Lifestyles for Baby (WEE Baby) Care Study aimed to integrate and coordinate care on responsive parenting (RP) guidance related to feeding, sleep, and play. The intervention was based on an evidence-based program that included messages that mothers believe should be a part of pediatric or WIC care [17-19]. The objective of this paper is to demonstrate the usability of advanced HIT strategies designed to engage parents in reporting RP practices, integrate data into electronic records to inform and facilitate documentation of provided RP care, and share data between settings to facilitate coordination of care between PCPs and WIC. The findings are intended to inform and advance novel cross-sector delivery models conceptualized to improve patient-centered care and health outcomes. While health outcomes are beyond the scope of this paper, these findings will inform the feasibility of models that align organizational resources and integrate activities for collective impact on population health objectives.

## Methods

### Study Design

The integrated and care coordination delivery model was constructed for the WEE Baby Care Study, which has been described elsewhere [20]. Briefly, the WEE Baby Care Study was a pragmatic trial to modify maternal parenting practices, with the goal of preventing rapid infant weight gain and obesity. Mothers and their newborn infants were assigned to either a 6-month RP intervention group (n=131) that included advanced HIT strategies to integrate and coordinate care between pediatric PCPs and WIC nutritionists or a control group (n=157) receiving standard care [20]. This analysis is limited to the 131 mother-infant dyads who were enrolled in the intervention group in which advanced HIT strategies were applied to collect patient-reported data, use these data to inform patient-centered care, securely share data, and coordinate care between PCPs and WIC nutritionists.

### Participants and Recruitment

From July 2016 to May 2018, mother-infant dyads were recruited and enrolled from northeastern Pennsylvania, an area characterized by the Health Services and Resources Administration as medically underserved, with shortages in health, dental, and mental health professionals [21]. Mother-infant dyads were either recruited in person in the labor and delivery unit or on the phone after the first well-child visit (WCV). Eligible dyads met the following inclusion criteria: full-term (≥37 weeks gestation) infant, singleton newborn, English-speaking mother between 18 and 55 years old, intention to receive well-child care at a participating pediatric clinic, and eligibility to be enrolled in or current enrollment in WIC. Participants provided written informed consent. All study procedures were approved by the institutional review boards of The Pennsylvania State University and Geisinger.

### Intervention and Advanced HIT Strategies

Mother-infant dyads randomized into the intervention group completed the Early Healthy Lifestyles (EHL) risk assessment tool prior to each WCV. The study team developed the EHL risk assessment tool to facilitate mothers’ self-assessments of their RP practices related to feeding, soothing, and playing with the infant, as well as the infant’s sleep behaviors. The parent was prompted by electronic messaging to complete the EHL in the patient portal prior to the WCV. The mother’s proxy access to the child’s portal was ensured at study enrollment. If the parent did not complete the tool, clinic staff encouraged the parent to complete the EHL in the waiting room on a tablet. Parent responses were then instantly integrated into the infant’s electronic health record (EHR) (Epic Systems).

Within the WCV progress note, PCPs could view and use the EHL data to inform and document patient-centered RP care. Prior to the start of the intervention, participating PCPs were trained to evaluate the EHL data, deliver tailored RP messages that aligned with parent learning needs at WCVs, and document provided RP care in the study-specific table in the WVC progress note, herein referred to as the PCP system. Age-appropriate, preventive messages were informed by the Intervention Nurses Start Infants Growing Healthy Trajectories (INSIGHT) study and American Academy of Pediatrics Healthy Active Living for Families curriculum [17,22,23]. PCPs used progress note functions to facilitate documentation of RP care provided during the WCV. Additionally, PCPs could add an 80-character, free-text comment to send to the WIC nutritionist, perhaps to direct attention to a high-priority issue. No action by the PCP was required to initiate data sharing. Development of the PCP system was guided, approved, and integrated into the EHR by Geisinger’s Health Information Technology Team.

**Figure 1:**
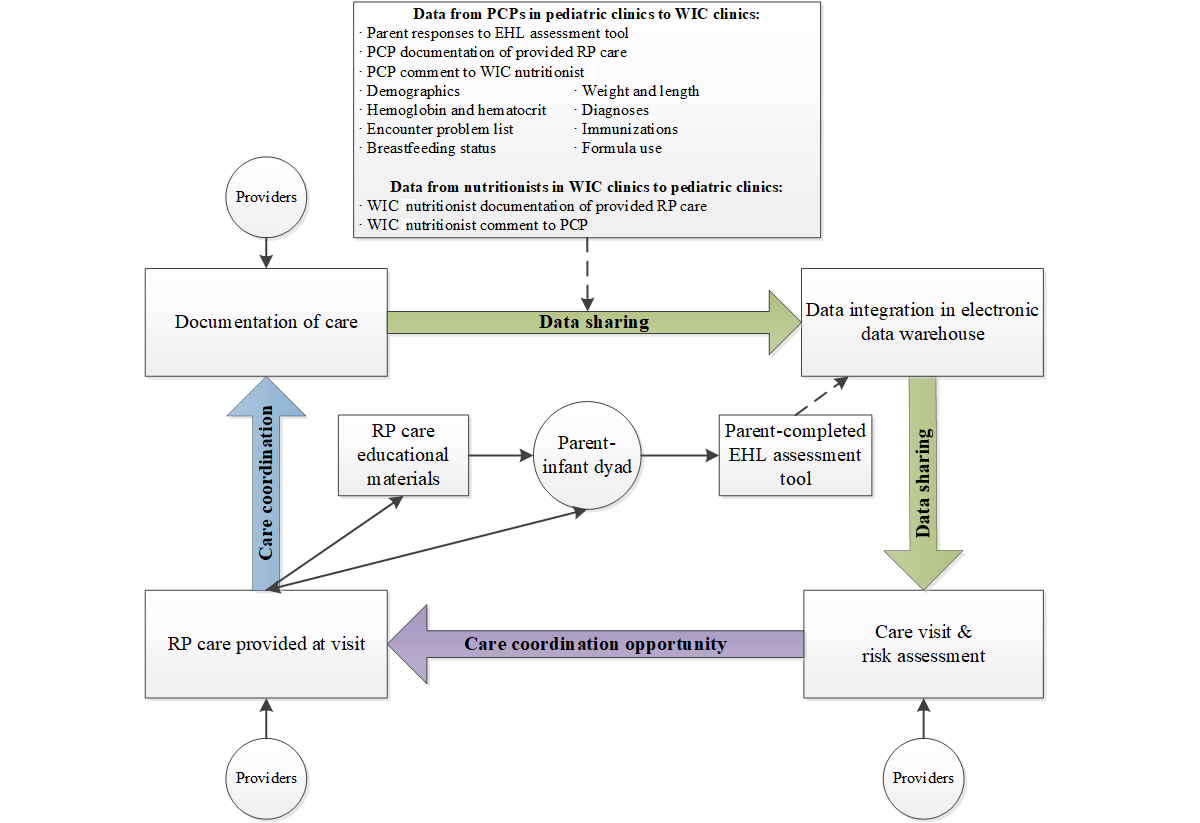
WEE Baby Care Study data flow for collection from parents, use by providers, and sharing for care coordination between clinic and community settings. EHL: Early Healthy Lifestyles; PCP: primary care provider; RP: responsive parenting; WIC: Special Supplemental Nutrition Program for Women, Infants, and Children.

Advanced HIT strategies were used to automate and securely share data of interest (Textbox 1) from the PCP to WIC, including parent EHL responses, PCP documentation of RP care, and child physical assessment. In turn, these data were automatically integrated and displayed in a study-specific system (herein referred to as the WIC system) to inform patient-centered counseling as coordinated care in the community setting. The WIC system was separate from WIC’s standard electronic participant management system—QuickWIC, a HTML system developed in the late 1990s—and could be used in parallel. Separate log-ins were required for each system. WIC nutritionists documented RP care in QuickWIC and comments in the WIC system. The WIC system extracted education codes from QuickWIC. Following the visit with the trained WIC nutritionist, encounter data of interest (Textbox 1) were extracted from the WIC system and securely shared from WIC to the PCP, thus providing a communication feedback loop to coordinate care at subsequent visits. Uniquely, the WIC system required the nutritionist to sign off on the record after the visit was completed to initiate data sharing. WIC nutritionists received training on this process, the use of EHL data, and the delivery of RP care messages prior to the start of the intervention. A booster training session was provided in the 14th month of the intervention. Data were exchanged between the PCP system and the WIC system through a secure file transport portal managed by the clinical care setting study team, and data were refreshed on weekdays. This process continued for a 6-month period to allow multiple opportunities for care coordination using shared data across clinic and community settings. Figure 1 provides an overview of the flow for patient-reported data collection, integration, sharing, and care coordination. An application developer at the Pennsylvania Department of Health developed and implemented the WIC system as an external cloud system as opposed to an integrated system, as statewide plans were underway to replace the QuickWIC system.

Attendance of WCVs and WIC visits was not mandatory or incentivized in this pragmatic study but was necessary for data of interest to be collected. The typical WCV schedule for infants within the health system includes visits at 3 to 5 days (newborn), 1 month, 2 months, 4 months, and 6 months of age. The study team expected that the intervention participants would attend at least 4 WCVs. To receive WIC benefits, infants and their mothers enrolled in WIC need to have a visit every 3 months; thus, the study team expected intervention participants would attend at least 2 WIC visits. In sum, this would allow multiple opportunities for care coordination between PCPs and WIC.

**Existing and study-specific systems with associated data elements shared by pediatric primary care providers from well-child visits and the Special Supplemental Nutrition Program for Women, Infants, and Children (WIC) nutritionists from appointments to inform care coordination. Italics indicate essential data elements for data set used for coding of data sharing, care coordination opportunity, and care coordination. Infant date of birth and enrollment date were extracted from the research team’s records.**

**Data from pediatric clinics**

Existing electronic health record:

- *Date of well-child visit*
- *Weight and length*
- *Immunizations*
- Demographics
- Hemoglobin and hematocrit
- Encounter and problem list
- Diagnoses
- Breastfeeding status
- Formula use

Study-specific primary care provider system integrated into electronic health record:

- *Parent completion of Early Healthy Lifestyles risk assessment*
- *Date that Early Healthy Lifestyles risk assessment was completed by parent*
- *Primary care provider documentation of Early Healthy Lifestyles preventive counseling*
- *Primary care provider comment to WIC nutritionist*

**Data from WIC clinics**

Existing QuickWIC system:

- *Date of WIC visit*
- *Date that nutritionist documented responsive parenting preventive care*
- *Nutritionist documentation of responsive parenting preventive care. The WIC nutritionist documented responsive parenting preventive care with standard WIC topic codes, as well as codes developed for the study.*

Study-specific WIC system:

- *Nutritionist comment to primary care provider*
- *Nutritionist sign-off on record*
- *Date of nutritionist sign-off*

### Outcome Definitions

The outcomes of interest included the use of the advanced HIT strategies and the feasibility of data sharing and care coordination occurring in 2 directions, from PCPs to WIC and from WIC to PCPs*.* Data sharing is the electronic and systematic push of study-related data through the electronic data warehouse (distinct from the EHR and PCP system) managed by the research team in the clinical setting. Data sharing does not require the PCP or WIC nutritionist in the receiving setting to interact with the data of interest (Textbox 1).

A care coordination opportunity is nested within data sharing and is an opportunity for a PCP or WIC nutritionist to view data for the most recent visit from the sending setting. Due to the secure and study staff–dependent data sharing processes, the receiving provider was able to view the shared data within 2 business days if sent by the PCP and 1 business day if sent by the WIC nutritionist. Eligible WCVs occurred after the dyad was enrolled into the study and continued up to 6 months of the infant’s age, at which time the clinical study team turned off the PCP system. Eligible WIC visits occurred after the dyad was enrolled in the study but before disabling the PCP system.

Care coordination is nested within a care coordination opportunity and requires a PCP or WIC nutritionist in the receiving setting to be able to access the sent data and document the provided RP care. The study team could not directly verify that the receiving setting interacted with the sent data, as accessing or viewing the data was not captured with a discrete data point; thus, the documentation of RP care serves as proxy evidence that the receiving provider used the shared data to coordinate care. Preventive RP care included educational messages that addressed parent learning needs as self-assessed by parents using the EHL tool. Preventive RP care was documented by PCPs and WIC nutritionists by selecting RP topics, writing a free-text comment, or both.

### Coding of Outcomes

The coding process was operationally detailed in a study team–developed coding manual that was extensively reviewed multiple times by 5 study team members (SMRK, HAH, MM, JSS, and LBD) and applied to a comprehensive, single data set. The data set included data from the PCP system and the WIC system along with other data elements listed in Textbox 1 to identify data sharing, care coordination opportunities, and care coordination outcomes. Two postdoctoral-level study team members (SMRK and HAH) coded the data independently, discrepancies were discussed and resolved using the coding manual, and both team members recoded the discrepant observations. After the first round of coding, 86% of coded observations agreed between the two coders, demonstrating adequate understanding of the coding process. This process continued until the 2 sets of coded data completely matched.

**Figure 2:**
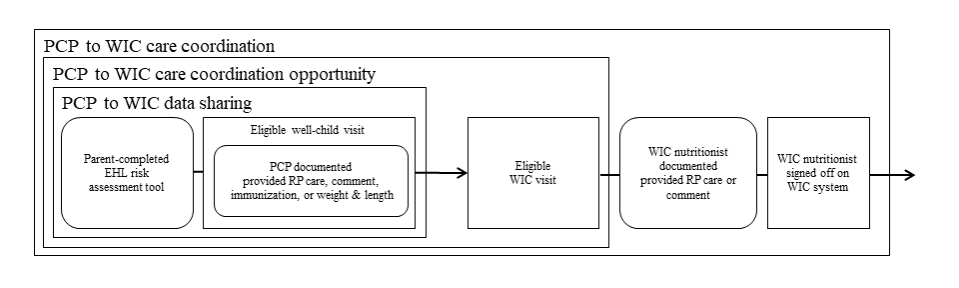
Components of data sharing, care coordination opportunities, and care coordination from pediatric PCPs to WIC nutritionists. EHL: Early Healthy Lifestyles; PCP: primary care provider; RP: responsive parenting; WIC: Special Supplemental Nutrition Program for Women, Infants, and Children.

PCP to WIC data sharing, care coordination opportunities, and care coordination events were identified. The criteria for these outcomes are depicted in Figure 2. PCP to WIC data sharing required an eligible WCV to have at least one of the following data points documented: (1) parent-completed EHL, (2) documentation of RP care provided by the PCP, (3) PCP comment to WIC, (4) immunization records, or (5) weight and length. After the identified PCP to WIC data sharing occurrence, a PCP to WIC care coordination opportunity occurred when the WCV was followed by an eligible WIC visit that occurred two or more business days after the WCV to allow ample time for the shared data to be available in the WIC system for the nutritionist. Subsequently, a PCP to WIC care coordination event was identified when the WIC nutritionist documented provided RP care or commented to the PCP and signed off on the record.

**Figure 3:**
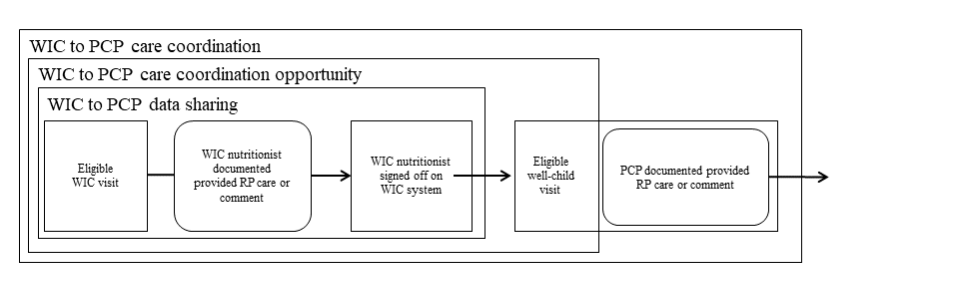
Components of data sharing, care coordination opportunities, and care coordination from WIC nutritionists to pediatric PCPs. PCP: primary care provider; RP: responsive parenting; WIC: Special Supplemental Nutrition Program for Women, Infants, and Children.

WIC to PCP data sharing, care coordination opportunities, and care coordination events were identified. As shown in Figure 3, WIC to PCP data sharing occurred when an eligible WIC visit had (1) documentation of RP care provided by the nutritionist or (2) a nutritionist comment or comments to the PCP, in addition to (3) a nutritionist sign-off on the same day or after the WIC visit. After the identified WIC to PCP data sharing occurrence, a WIC to PCP care coordination opportunity was identified when the WIC sign-off date was directly followed by a WCV to allow for shared data to be available in the PCP system for the PCP. Subsequently, a WIC to PCP care coordination event was identified when the PCP documented EHL-related preventive counseling or commented to WIC on the same day as the WCV.

### Statistical Analysis

All data were processed and analyzed using SAS (version 9.4; SAS Institute Inc). The SAS functions “Proc Freq” and “Proc Means” were used to describe the sample and determine the total number and mean per dyad (with standard deviation) of variables of interest to describe the usability of the advanced HIT technologies and feasibility of data sharing and care coordination using the coded variables described above.

## Results

### Participants

As shown in Table 1, 49.6% (65/131) of infants were male and 67.7% (88/131) were White. Mothers were aged 27.7 (SD 5.7) years at the time of infant delivery, and 26.0% (34/131) were primiparous. Infants were enrolled at 0.34 (SD 0.43) months of age. Most mothers were White, single, had an income less than $50,000, and had at least a high school diploma.

**Table 1:** Demographic characteristics of mothers and infants who were assigned to a parenting intervention (N=131) that used advanced health information technology strategies to integrate and coordinate care in the WEE Baby Care Study.

Demographic characteristic			Responsive parenting intervention mother-infant dyads (N=131)
**Infant**			
	Male, n (%)		65 (49.6)
	Gestational age (weeks), mean (SD)		39.7 (1.1)
	Birth weight (kg), mean (SD)		3.47 (0.43)
	Birth length (cm), mean (SD)		49.2 (2.3)
	Age at enrollment (months), mean (SD)		0.34 (0.43)
	**Infant race, n (%)**		
		Black	22 (16.9)
		White	88 (67.7)
		American Indian or Alaskan Native	0 (0.0)
		Asian	1 (0.8)
		Other	19 (14.6)
**Mother**			
	Age at infant birth (years), mean (SD)		27.7 (5.7)
	Diabetes during pregnancy, n (%)		20 (16.1)
	Smoked during pregnancy, n (%)		26 (21.0)
	Hispanic, n (%)		26 (21.1)
	Primiparous, n (%)		34 (26.0)
	**Marital status, n (%)**		
		Married	25 (20.2)
		Not married, living with partner	34 (27.4)
		Single	57 (46.0)
		Divorced or separated	6 (4.8)
		Widowed	0 (0.0)
		Other	2 (1.6)
	**Annual household income (US $),** **n (%)**		
		<10,000	28 (22.6)
		10,000-24,999	52 (41.9)
		25,000-49,999	30 (24.2)
		50,000-74,999	3 (2.4)
		Do not know	8 (6.5)
		Refuse to answer	3 (2.4)
	**Education, n (%)**		
		Some high school or less	13 (10.5)
		High school graduate	60 (48.4)
		Some college	41 (33.1)
		College graduate	9 (7.3)
		Graduate degree or greater	1 (0.8)

### Use of Advanced HIT Strategies

The 131 intervention mother-infant dyads attended 459 eligible WCVs and 296 eligible WIC visits throughout the observation period; thus, participants attended over 3 (mean 3.5, SD 1.03) WCVs per dyad on average and over 2 (mean 2.26, SD 0.97) WIC visits per dyad on average (Table 2). Of the expected 4 WCVs and 2 WIC visits per dyad to be experienced in the observation period, 45.8% (60/131) of dyads attended 4 or more WCVs and 78.6% (103/131) attended 2 or more WIC visits.

Mothers completed the EHL risk assessment 262 times (mean 2.0, SD 1.23) (Table 3). Of the expected EHL completions prior to a WCV, 10.7% (14/131) of dyads completed 4 or more EHL risk assessments, but 16.0% (21/131) did not complete a single EHL assessment.

PCPs used the PCP system to document RP care at least once (mean 1.37, SD 1.30) per participant but almost never wrote a comment to the nutritionist (mean 0.02, SD 0.15 comments per participant). Of the expected 4 or more WCVs, PCPs documented RP care for 6.9% (9/131) of dyads, but 35.1% (46/131) of participants did not have any RP care documented by the PCP. Anthropometric measures and immunization data were routinely available in the infants’ EHR (Table 4).

Nutritionists documented RP care at least twice per participant (mean 2.21, SD 0.82) (Table 3). Nutritionist comments to PCPs were provided at least once per participant (mean 1.5, SD 0.96). Of the expected 2 or more WIC visits, nutritionists documented RP care for 81.6% (107/131) of dyads and comments for 46.5% (61/131) of dyads; 1.5% (2/131) and 14.5% (19/131) of dyads did not any have documentation of RP care or a nutritionist comment to the PCP, respectively. Nutritionists used the WIC system to sign off on a single record per participant (mean 1.64, SD 1.03). Of the expected 2 or more WIC visits, slightly over half of dyads (71/131, 54.1%) had a sign-off from the nutritionist in the WIC system, but 32.8% (43/131) and 13.0% (17/131) of participants only had 1 or no sign-offs, respectively, which prevented WIC to PCP data sharing and data coordination opportunities.

**Table 2:** Total number and average per participant of data sharing, care coordination opportunities, and care coordination events between clinic and community settings for 131 mother-infant dyads in the WEE Baby Care Study.

Event			Totals for intervention sample (N=131)	Descriptive statistics for intervention participants	
			Events, n (%)	Mean (SD)	Range
Clinic WCV^a,b^			459	3.50 (1.03)	1-7
Community WIC^c,d^ visits			296	2.26 (0.97)	0-5
**PCP to WIC data sharing and care coordination**					
	**PCP to WIC data sharing**		459	3.50 (1.03)	1-7
		with length and weight measures	457 (99.6)	3.49 (1.02)	1-7
		with immunization records	231 (50.3)	1.76 (0.70)	0-3
		with Early Healthy Lifestyles risk assessment	244 (53.2)	1.86 (1.15)	0-4
		with PCP^e^ documentation of provided RP^f^ care	162 (35.3)	1.24 (1.19)	0-4
		with PCP comment to WIC nutritionist	3 (0.7)	0.02 (0.15)	0-1
	**PCP to WIC care coordination opportunity**		231	1.76 (0.81)	0-3
		with length and weight measures	231 (100.0)	1.76 (0.81)	0-3
		with immunization records	137 (59.3)	1.05 (0.63)	0-3
		with Early Healthy Lifestyles risk assessment	134 (58.0)	1.02 (0.86)	0-3
		with PCP documentation of provided RP care	88 (38.1)	0.67 (0.72)	0-3
		with PCP comment to WIC nutritionist	1 (0.4)	0.008 (0.09)	0-1
	**PCP to WIC care coordination**		154	1.18 (0.85)	0-3
		with length and weight measures	154 (100.0)	1.18 (0.85)	0-3
		with immunization records	86 (55.8)	0.66 (0.85)	0-2
		with Early Healthy Lifestyles risk assessment	84 (54.5)	0.64 (0.76)	0-3
		with PCP documentation of provided RP care	57 (37.0)	0.44 (0.61)	0-3
		with PCP comment to WIC nutritionist	1 (0.6)	0.008 (0.09)	0-1
		with WIC documentation of provided RP care	153 (99.4)	1.15 (0.84)	0-3
		with WIC nutritionist comment to PCP	144 (93.5)	1.10 (0.81)	0-3
**WIC to PCP data sharing and care coordination**					
	**WIC to PCP data sharing**		197	1.50 (0.95)	0-4
		with WIC documentation of provided RP care	193 (98.0)	1.47 (0.91)	0-4
		with nutritionist comment to PCP	183 (92.9)	1.40 (0.91)	0-4
	**WIC to PCP care coordination opportunity**		116	0.89 (0.78)	0-3
		with WIC documentation of provided RP care	114 (98.3)	0.87 (0.78)	0-3
		with nutritionist comment to PCP	106 (91.4)	0.81 (0.73)	0-3
	**WIC to PCP care coordination**		51	0.39 (0.58)	0-2
		with WIC documentation of provided RP care	50 (98.0)	0.38 (0.56)	0-2
		with nutritionist comment to PCP	48 (94.1)	0.37 (0.56)	0-2
		with PCP documentation of provided RP care	51 (100.0)	0.39 (0.58)	0-2
		with PCP comment to WIC nutritionist	1 (2.0)	0.008 (0.09)	0-1

^a^WCV: well-child visit.

^b^Of the expected 4 WCVs to be attended by the dyad in the intervention period, 45.8% (60/131) of dyads attended 4 of more WCVs.

^c^WIC: Special Supplemental Nutrition Program for Women, Infants, and Children.

^d^Of the expected 2 WIC visits to be attended by the dyad in the intervention period, 77.9% (103/131) of dyads attended 2 or more WIC visits.

^e^PCP: primary care provider.

^f^RP: responsive parenting.

**Table 3:** Total number and average per individual participant of intervention components derived from clinical and community settings for 131 mother-infant dyads in the responsive parenting intervention arm of the WEE Baby Care Study.

Intervention component		Frequency for total sample (N=131)	Descriptive statistics for intervention participants	
		Total Events, n	Mean (SD)	Range
**Clinical intervention components from WCV^a^**				
	Infant length and weight measures	665	5.08 (2.01)	1-15
	Immunization records	296	2.26 (0.83)	0-4
	Early Healthy Lifestyles (EHL) risk assessment	262	2.00 (1.23)	0-5
	PCP^b^ documentation of provided RP^c^ care	180	1.37 (1.30)	0-4
	PCP comment to WIC nutritionist	3	0.02 (0.15)	0-1
**Community intervention components from WIC^d^**				
	Nutritionist documentation of provided RP care	289	2.21 (0.82)	0-4
	Nutritionist comment to PCP	196	1.50 (0.96)	0-4
	Nutritionist signed off in WIC system^e^	215	1.64 (1.03)	0-6

^a^WCV: well-child visit.

^b^PCP: primary care provider.

^c^RP: responsive parenting.

^d^WIC: Special Supplemental Nutrition Program for Women, Infants, and Children.

^e^WIC system: study-specific system that allowed nutritionists to sign off on study activity to share data.

**Table 4:** Frequency distributions of intervention components derived from clinical and community settings for 131 mother-infant dyads in the responsive parenting intervention arm of the WEE Baby Care Study.

Component		Number of times event occurred, n (%)							
		0 times	1 time	2 times	3 times	4 times	5 times	6 times	7+ times
**Clinical intervention components from WCV^a^**									
	Length and weight measures	1 (0.7)	6 (4.6)	13 (9.9)	41 (31.3)	27 (20.6)	18 (13.7)	11 (8.4)	14 (10.7)
	Immunization records	3 (2.3)	15 (11.5)	66 (50.4)	39 (29.8)	8 (6.1)	0 (0.0)	0 (0.0)	0 (0.0)
	Early Healthy Lifestyles risk assessment	21 (16.0)	20 (15.3)	43 (32.8)	33 (25.2)	13 (9.9)	1 (0.7)	0 (0.0)	0 (0.0)
	PCP^b^ documentation of provided RP^c^ care	46 (35.1)	30 (22.9)	24 (18.3)	22 (16.8)	9 (6.9)	0 (0.0)	0 (0.0)	0 (0.0)
	PCP comment to WIC nutritionist	128 (97.7)	3 (2.3)	0 (0.0)	0 (0.0)	0 (0.0)	0 (0.0)	0 (0.0)	0 (0.0)
**Community intervention components from WIC^d^**									
	Nutritionist documentation of provided RP care	2 (1.5)	22 (16.8)	59 (45.0)	43 (32.8)	5 (3.8)	0 (0.0)	0 (0.0)	0 (0.0)
	Nutritionist comment to PCP	19 (14.5)	51 (38.9)	39 (29.8)	21 (16.0)	1 (0.7)	0 (0.0)	0 (0.0)	0 (0.0)
	Nutritionist signed off in WIC system^e^	17 (13.0)	43 (32.8)	45 (34.4)	24 (18.3)	1 (0.7)	1 (0.7)	0 (0.0)	0 (0.0)

^a^WCV: well-child visit.

^b^PCP: primary care provider.

^c^RP: responsive parenting.

^d^WIC: Special Supplemental Nutrition Program for Women, Infants, and Children.

^e^WIC system: study-specific system that allowed nutritionists to sign off on study activity to share data.

### Feasibility of Data Sharing and Care Coordination

PCP to WIC data sharing occurred for all 459 (100%) WCVs (Table 2). Almost all events (257/259, 99.6%) included the infant’s length and weight, and half included immunization records (231/459, 50.3%) and parent responses to EHL assessment (244/459, 53.2%). One-third of PCP to WIC data sharing events (162/459, 35.3%) included documentation of PCP RP care; thus, one event per dyad had PCP documentation (mean 1.24, SD 1.19). Very few events (3/459, 0.7%) had a PCP comment to the WIC nutritionist. A WIC visit followed 231 of the 459 (50.3%) PCP to WIC data sharing events; thus, dyads averaged less than 2 (1.7, SD 0.81) PCP to WIC care coordination opportunities (Table 2). All (231/231, 100%) PCP to WIC care coordination opportunities included the infant’s length and weight, slightly more than half included immunization records (137/231, 59.3%) and EHL responses (134/231, 58.0%), and about one-third (88/231, 38.1%) included PCP documentation of RP care.

PCP to WIC care coordination was evident in 154 of 231 (66.7%) of PCP to WIC care coordination opportunities; thus, dyads averaged at least one PCP to WIC care coordination event (1.18, SD 0.85) (Table 2). WIC nutritionists were able to view anthropometric measures at all care coordination events (154/154, 100%), immunization records at 55.8% (86/154) of events, parent EHL responses at 54.5% (84/154) of events, and PCP documentation of RP care at 37.0% (57/154) of care coordination events. Dyads, on average, had few events that included their EHL responses (mean 0.64, SD 0.76) and PCP documentation of RP care (mean 0.44, SD 0.61), thus limiting opportunities for nutritionists to use EHL data and PCP RP care to inform care and coordinate with PCPs. To coordinate care, however, the trained WIC nutritionists documented RP counseling (153/154, 99.4%) and commented (144/154, 93.5%) at nearly all events; therefore, on average, each dyad received EHL-related RP preventive counseling from a WIC nutritionist one time (1.15, SD 0.84). Of the 231 care coordination opportunities, the 77 missed opportunities were primarily due to a missing record sign-off, as WIC nutritionists consistently documented RP care.

Given the bidirectional flow of care and data, intervention components and outcomes were also evaluated from WIC to PCPs as events independent from PCP to WIC care coordination*.* Data sharing from WIC to PCPs occurred for 197 of 296 (66.6%) WIC visits (Table 2). Nearly all WIC visits included documented EHL-related preventive counseling (193/197, 98.0%) or comments to the PCP (183/197, 92.9%). A WCV followed 116 of the 197 (58.9%) WIC to PCP data sharing events; thus, dyads averaged just under 1 (0.89, SD 0.78) WIC to PCP care coordination opportunity. The majority of WIC to PCP care coordination opportunities included documentation of RP preventive counseling (114/166, 98.3%) and comments to the PCP (106/166, 91.4%). At 51 of 116 (44.0%) WIC to PCP care coordination opportunities, PCPs documented RP preventive counseling (51/51, 100%) or a comment (1/51, 2.0%) to the nutritionist to coordinate care; thus, dyads averaged few (0.39, SD 0.58) WIC to PCP care coordination events. PCPs were able to view nutritionist documentation of RP preventive counseling (50/51, 98.0%) and comments (48/51, 94.1%).

## Discussion

This study suggests that advanced HIT strategies are a potential solution to engage parents in reporting RP practices, integrate data into the infant’s electronic patient management systems to inform and facilitate documentation of provided RP care, and share data between PCPs and WIC nutritionists serving socioeconomically disadvantaged parents and infants from an area with known shortages in health care services. Even though bidirectional data sharing was feasible, care coordination occurred less frequently. Typically, these advanced HIT strategies are commonly found and siloed in larger health systems with standardized EHRs, a resource supported by policy and practice [11-13]; however, the current study applied advanced HIT strategies to bidirectionally share data across local community and clinical settings, an important step in coordinating patient-centered preventive care. Implemented strategies used to collect, integrate, and share data were adopted by patients, PCPs, and WIC nutritionists, suggesting usability, but variable utilization of certain components inhibited care coordination. Replication and dissemination of a cross-sector model may be facilitated by clinical partners practicing in a standardized EHR environment, with these findings providing foundational lessons and recommendations for future iterations.

Observed data sharing between clinic and community settings demonstrated usability, yet PCP to WIC data sharing occurred at more than twice the rate of WIC to PCP data sharing, likely mirroring the more frequently scheduled and attended WCVs relative to WIC visits. The infrastructure and processes developed to share data between PCPs and WIC differed in reliability and efficiency. PCP to WIC data sharing functioned more reliably than in the reverse direction. Data elements were passively extracted from EHRs without PCP action beyond standard documentation and thus were reliably (459/459, 100%) shared with WIC after the WCVs. In comparison, WIC to PCP data sharing was less reliable (197/296, 66.6%), as the process required the WIC nutritionist to sign off in the WIC system, a step that was missed for one-third of events, impacting usability. In addition, this extra step in the nutritionists’ workflow was critical for PCP to WIC care coordination, as a sign-off on the WIC system was required to complete the coordination process with the PCP. This suggests that processes that change standard workflow or require an additional step may inhibit data sharing from the community setting. However, passive HIT strategies to collect and extract data can feasibly and effectively facilitate data sharing from a clinical to a nonclinical setting; thus, future iterations of this model should use passive, automatic processes in both settings.

Although WIC to PCP data sharing was less effective, the process was more efficient than in the reverse direction. The WIC to PCP data sharing process took 1 business day and was dependent on human resources. After the WIC nutritionist signed off of the WIC system, data were extracted that evening and sent to the research team in the clinical setting via a secure file transport portal and then integrated into the infant’s EHR by a research team staff member the next morning. In contrast, after data were extracted from the EHR the morning after a WCV, data were sent via a secure file transport portal to a centralized team member at WIC, who uploaded the data to the WIC system, which refreshed at midnight via an automatic batch process. Thus, the process for PCP to WIC data sharing was 2 business days and was also human dependent. Due to staff availability in both the clinical research and central WIC settings, all data transfer steps were delayed by holidays and staff absences, planned and unplanned. Even though the data sharing process between PCPs and WIC was completed in 1 to 2 business days, future applications should aim to shorten this delay and automate the data transfer in real time. Addressing these limitations would accommodate participants who attend multiple visits within the same day or on consecutive days and reduce reliance on human resources, thus increasing the generalizability of the model to real-world settings with limited resources.

Care coordination was seldomly observed, in part due to the limitations of the data sharing processes, the wide variation in the attendance (frequency and pattern) of both WCVs and WIC visits, and the limited adoption of the PCP system to document RP care. Aside from logistical data sharing issues, enrollment of dyads after their first WCV or WIC visit, along with the sequence in which they attended visits throughout the study, limited opportunities for care coordination. However, when WIC nutritionists had the opportunity, documentation of RP care by the WIC nutritionists was high. Overall, dyads had 2 events with nutritionist documentation of RP care, which aligned with the 2 WIC visits attended. Almost all (193/197, 98.0%) of WIC visits with data shared to PCPs included documentation of this RP care. Nutritionists wrote a free-text comment to the PCP at a similarly high rate. In contrast, fewer participant records had evidence of PCP documentation of RP care, and PCPs only wrote a comment to WIC a total of 3 times. Overall, dyads had 1 event with PCP documentation of RP care despite attending 3 WCVs. Therefore, WIC nutritionists readily documented RP care and used the free-text comment feature, but adoption of this feature by PCPs was more variable.

Low adoption of the documentation features of the PCP system may be due to variations in PCP practice that limited exposure to the system. Infants were randomized at the individual level as opposed to the PCP or clinic level. Thus, a PCP provided well-child care to infants in the treatment group (with the PCP system) and control group (without the PCP system), as well as to infants not involved in the study (without the PCP system) throughout the 22-month study period, creating inconsistencies in workflow and exposure to the PCP system. Out of the 459 WCVs attended by intervention dyads, PCPs documented RP preventive education at 162 (35.3%) visits. Documentation of RP preventive education, however, was a proxy for PCP system use and did not capture if the PCP viewed the EHL assessment tool or data from WIC within the PCP system and documented education elsewhere, such as the WCV note. Adoption of interventional EHR components may be improved through randomization at the PCP level, longer exposure times to innovations, additional training and booster sessions, feedback on performance, and enhanced organizational factors, such as administrative and operational support [24,25].

High adoption of RP care by WIC nutritionists may reflect strong alignment with their focused program goals and requirements [1]. This is evidenced by the high frequency of documented RP care in participant WIC records. Documentation of RP care was facilitated by using standard WIC education codes and integrating study team–created codes into WIC’s standard electronic system (QuickWIC). Further, at some WIC visits, nutritionists could use parent-reported EHL data available to inform care. However, parent completion of EHL and the required cadence for a PCP to WIC care coordination opportunity (ie, a WCV followed by a WIC visit) limited the availability of EHL data to the nutritionist to a single event per dyad. Even though EHL data were available at about half of WIC visits, nutritionists provided and documented EHL-related RP education at most visits with dyads. In comparison, PCPs comprehensively assess and address growth, development, and safety issues at WCVs but perceive WIC nutritionists as having more time to discuss important nutrition and feeding issues and predicted clear benefits in cross-sector integration and coordination [4]. Thus, sharing nutritionists’ documentation of preventive counseling may provide opportunities for the PCP to coordinate care by allowing the brief reinforcement of messages or time to discuss new or other topics pertinent to the care of their patient.

The study demonstrated that HIT strategies can facilitate the collection, integration, and sharing of patient-reported data on parenting practices in 2 settings. Most parents routinely completed the EHL 2 or more times, and PCPs had access to parent-reported EHL data at about half of the visits, which was then shared with WIC nutritionists to view at more than half of the care coordination opportunities. Parent responses to the EHL were automatically integrated into the infant’s electronic health record in the pediatric setting and into the study-specific WIC system to prompt PCPs and WIC nutritionists to provide tailored RP counseling. Integrating parent-reported data into health care professionals’ workflow and electronic patient management system prior to a visit may lead to patient-centered care by highlighting timely concerns and streamlining patient assessment. However, electronic solutions to collect patient-reported information need to account for technology availability at both the patient and clinic levels so as to not exacerbate the digital divide seen between lower- and higher-income populations [26].

Reliance on human resources for data sharing impacted the reliability and efficiency of processes to transfer data between clinical and community settings to facilitate care coordination; however, the system developed for this study addressed stakeholder concerns related to security of data sharing across settings [4]. Importantly, given the imperative of protecting patient rights and the health system’s responsibility to maintain privacy, patient consent to share a limited data set across settings is warranted [4,16,27]. Executing interinstitutional agreements between the community and clinical settings for limited data sharing with participant consent was a critical and substantial step in the development of data sharing processes and addressed stakeholder concerns. In addition, using in-person or over-the-phone staff recruitment to obtain participant consent using paper or electronic consent forms, as well as the use of a secure file transport portal, addressed privacy concerns related to the Health Insurance Portability and Accountability Act and data security [4]. Further innovations in HIT strategies are needed to more efficiently and effectively share limited patient data sets with aligned community and public health agencies to address limitations related to workflow, human resources, and timeliness. Within the clinical sector, HIT strategies have been used to facilitate efficient data exchange between providers, and technologies are emerging for data exchange between clinical and public health settings [28,29]. Pairing these HIT technologies with strategies for data integration, as described here, presents a rich opportunity for scaling cross-sector data sharing into practice in order to implement recommendations from integrated and coordinated care models, achieving collective impact and advancing population health objectives [9,15].

While a strength of the pragmatic study is the observation of real-life patterns of care, a limitation to testing care coordination was that few participants completed the expected 4 WCVs or 2 WIC visits in a 6-month observation period. Visit attendance, timing, and the sequence of visit types was not determined or controlled by the study team. Socioeconomically disadvantaged parents and infants experience many barriers to attending WIC appointments, including lack of transportation or childcare, conflicting activities, negative feelings about nutrition education, and frequent relocation [30]. Research in the prenatal period suggests that providing low-income additional supports, such as case managers or outreach and community health care workers [31], may enhance efforts to coordinate care between clinical and community settings by promoting timely visit attendance. Although WEE Baby Care was informed by a formative qualitative phase that captured the perceptions of stakeholders, including PCPs, WIC nutritionists, and parents [4], the perspectives of these stakeholders on the implementation of the intervention components were not captured, which limited our understanding of how PCPs and nutritionists integrated the intervention into their workflow. Additional training booster sessions and a longer intervention period may have improved adoption of the PCP and WIC systems through reminders and increased exposure. Further, the limitations of the WIC system and iterations of this model may be optimized, as WIC has implemented an updated, nationally standardized participant management system since the completion of this study, providing an opportunity to improve the technology as well as the potential for evaluation and spread. Recruiting dyads after the first WCV likely reduced the number of potential opportunities for data sharing and care coordination. Lastly, most of the participating low-income mothers were White, all were English speaking, and most had a high school diploma or higher. This was representative of the Geisinger region, but generalizability to the more diverse national WIC and low-income populations with lower educational attainment may be limited [32,33].

Advanced HIT strategies can share data across clinical and community settings, even in a socioeconomically disadvantaged area with shortages in health care services; however, improvements in the usability of key strategies are needed to facilitate care coordination and increase generalizability to other settings. In alignment with models calling for integration and care coordination across settings [9,15], this intervention employed a comprehensive set of advanced HIT strategies, including patient (parent) access to their infant’s EHR (patient portal), patient (parent) electronic completion of a risk assessment that becomes integrated into their infant’s EHR, secure data sharing mechanisms, the ability for PCPs and WIC nutritionists to communicate securely, system analytics to manage preventive care, and interoperability between clinical and community data systems [11,16]. Future research is needed to address process limitations affecting the reliability and effectiveness of data sharing and care coordination and the suboptimal use of health care services. Integrating clinical and community health care services through electronic data sharing with advanced HIT strategies could be an integral approach to providing coordinated, patient-centered health care to low-income, socioeconomically disadvantaged populations.

## Appendix

Multimedia Appendix 1 CONSORT eHealth Checklist V 1.6.1.

## Abbreviations

EHL: Early Healthy Lifestyles
EHR: electronic health record
HIT: health information technology
INSIGHT: Intervention Nurses Start Infants Growing Healthy Trajectories
PCP: primary care provider
RP: responsive parenting
WCV: well-child visit
WEE Baby: Women, Infants, and Children Enhancements to Early Healthy Lifestyles for Baby
WIC: Special Supplemental Nutrition Program for Women, Infants, and Children
