# Supplementary material for: Advanced Health Information Technologies to Engage Parents, Clinicians, and Community Nutritionists in Coordinating Responsive Parenting Care: Descriptive Case Series of the Women, Infants, and Children Enhancements to Early Healthy Lifestyles for Baby (WEE Baby) Care Randomized Controlled Trial
Source: JMIR Pediatr Parent. 2020 Nov 24;3(2):e22121. doi: 10.2196/22121 (PMC7723742; doi:10.2196/22121)
Supplement: Multimedia Appendix 1 [file pediatrics_v3i2e22121_app1.pdf]

# CONSORT-EHEALTH (V 1.6.1) - Submission/Publication Form

The CONSORT-EHEALTH checklist is intended for authors of randomized trials evaluating web-based and Internet-based applications/interventions, including mobile interventions, electronic games (incl multiplayer games), social media, certain telehealth applications, and other interactive and/or networked electronic applications. Some of the items (e.g. all subitems under item 5 - description of the intervention) may also be applicable for other study designs.

The goal of the CONSORT EHEALTH checklist and guideline is to be
a) a guide for reporting for authors of RCTs,
b) to form a basis for appraisal of an ehealth trial (in terms of validity)

CONSORT-EHEALTH items/subitems are MANDATORY reporting items for studies published in the Journal of Medical Internet Research and other journals / scientific societies endorsing the checklist.

Items numbered 1., 2., 3., 4a., 4b etc are original CONSORT or CONSORT-NPT (non-pharmacologic treatment) items.
Items with Roman numerals (i., ii, iii, iv etc.) are CONSORT-EHEALTH extensions/clarifications.

As the CONSORT-EHEALTH checklist is still considered in a formative stage, we would ask that you also RATE ON A SCALE OF 1-5 how important/useful you feel each item is FOR THE PURPOSE OF THE CHECKLIST and reporting guideline (optional).

Mandatory reporting items are marked with a red *.
In the textboxes, either copy & paste the relevant sections from your manuscript into this form - please include any quotes from your manuscript in QUOTATION MARKS,
or answer directly by providing additional information not in the manuscript, or elaborating on why the item was not relevant for this study.

YOUR ANSWERS WILL BE PUBLISHED AS A SUPPLEMENTARY FILE TO YOUR PUBLICATION IN JMIR AND ARE CONSIDERED PART OF YOUR PUBLICATION (IF ACCEPTED).
Please fill in these questions diligently. Information will not be copyedited, so please use proper spelling and grammar, use correct capitalization, and avoid abbreviations.

DO NOT FORGET TO SAVE AS PDF _AND_ CLICK THE SUBMIT BUTTON SO YOUR ANSWERS ARE IN OUR DATABASE !!!

Citation Suggestion (if you append the pdf as Appendix we suggest to cite this paper in the caption):
Eysenbach G, CONSORT-EHEALTH Group
CONSORT-EHEALTH: Improving and Standardizing Evaluation Reports of Web-based and

Mobile Health Interventions
J Med Internet Res 2011;13(4):e126
URL: http://www.jmir.org/2011/4/e126/
doi: 10.2196/jmir.1923
PMID: 22209829

* Required

Your name *

First Last

Samantha M.R. Kling

Primary Affiliation (short), City, Country *

University of Toronto, Toronto, Canada

Stanford School of Medicine, Stanford, CA, US/

Your e-mail address *

abc@gmail.com

skling@stanford.edu

Title of your manuscript *

Provide the (draft) title of your manuscript.

Applying Advanced Health Information Technologies to Engage Parents, Clinicians, and Community Nutritionists to Coordinate Responsive Parenting Care

### Name of your App/Software/Intervention *

If there is a short and a long/alternate name, write the short name first and add the long name in brackets.

Responsive Parenting Intervention for the WEE

### Evaluated Version (if any)

e.g. "V1", "Release 2017-03-01", "Version 2.0.27913"

Your answer

### Language(s) *

What language is the intervention/app in? If multiple languages are available, separate by comma (e.g. "English, French")

English

### URL of your Intervention Website or App

e.g. a direct link to the mobile app on app in appstore (itunes, Google Play), or URL of the website. If the intervention is a DVD or hardware, you can also link to an Amazon page.

Your answer

### URL of an image/screenshot (optional)

Your answer

### Accessibility *

Can an enduser access the intervention presently?

- ○ access is free and open
- ○ access only for special usergroups, not open
- ○ access is open to everyone, but requires payment/subscription/in-app purchases
- ○ app/intervention no longer accessible
- ◉ Other: WIC System is no longer available, PCP System is still in use

### Primary Medical Indication/Disease/Condition *

e.g. "Stress", "Diabetes", or define the target group in brackets after the condition, e.g. "Autism (Parents of children with)", "Alzheimers (Informal Caregivers of)"

Obesity

### Primary Outcomes measured in trial *

comma-separated list of primary outcomes reported in the trial

infant growth and obesity; parental use of fooc

### Secondary/other outcomes

Are there any other outcomes the intervention is expected to affect?

usability, feasibility, frequency of data sharing and care coordination

Recommended "Dose" *

What do the instructions for users say on how often the app should be used?

- ( ) Approximately Daily
- ( ) Approximately Weekly
- ( ) Approximately Monthly
- ( ) Approximately Yearly
- ( ) "as needed"
- (x) Other: Used with standard pediatric well-child visits and WIC visits

Approx. Percentage of Users (starters) still using the app as recommended after 3 months *

- (x) unknown / not evaluated
- ( ) 0-10%
- ( ) 11-20%
- ( ) 21-30%
- ( ) 31-40%
- ( ) 41-50%
- ( ) 51-60%
- ( ) 61-70%
- ( ) 71%-80%
- ( ) 81-90%
- ( ) 91-100%
- ( ) Other:

Overall, was the app/intervention effective? *

- ( ) yes: all primary outcomes were significantly better in intervention group vs control
- ( ) partly: SOME primary outcomes were significantly better in intervention group vs control
- ( ) no statistically significant difference between control and intervention
- ( ) potentially harmful: control was significantly better than intervention in one or more outcomes
- ( ) inconclusive: more research is needed
- (•) Other: This manuscript is an exploratory analysis on the usability and feasibi

Article Preparation Status/Stage *

At which stage in your article preparation are you currently (at the time you fill in this form)

- ( ) not submitted yet - in early draft status
- ( ) not submitted yet - in late draft status, just before submission
- ( ) submitted to a journal but not reviewed yet
- (•) submitted to a journal and after receiving initial reviewer comments
- ( ) submitted to a journal and accepted, but not published yet
- ( ) published
- ( ) Other:

## Journal *

If you already know where you will submit this paper (or if it is already submitted), please provide the journal name (if it is not JMIR, provide the journal name under "other")

- ○ not submitted yet / unclear where I will submit this
- ○ Journal of Medical Internet Research (JMIR)
- ○ JMIR mHealth and UHealth
- ○ JMIR Serious Games
- ○ JMIR Mental Health
- ○ JMIR Public Health
- ○ JMIR Formative Research
- ◉ Other JMIR sister journal
- ○ Other:

## Is this a full powered effectiveness trial or a pilot/feasibility trial? *

- ○ Pilot/feasibility
- ◉ Fully powered

## Manuscript tracking number *

If this is a JMIR submission, please provide the manuscript tracking number under "other" (The ms tracking number can be found in the submission acknowledgement email, or when you login as author in JMIR. If the paper is already published in JMIR, then the ms tracking number is the four-digit number at the end of the DOI, to be found at the bottom of each published article in JMIR)

- ○ no ms number (yet) / not (yet) submitted to / published in JMIR
- ◉ Other: #22121

## TITLE AND ABSTRACT

## 1a) TITLE: Identification as a randomized trial in the title

### 1a) Does your paper address CONSORT item 1a? *

I.e does the title contain the phrase "Randomized Controlled Trial"? (if not, explain the reason under "other")

- ○ yes
- ◉ Other: This manuscript only analyzes data from the intervention arm of the 2

### 1a-i) Identify the mode of delivery in the title

Identify the mode of delivery. Preferably use "web-based" and/or "mobile" and/or "electronic game" in the title. Avoid ambiguous terms like "online", "virtual", "interactive". Use "Internet-based" only if Intervention includes non-web-based Internet components (e.g. email), use "computer-based" or "electronic" only if offline products are used. Use "virtual" only in the context of "virtual reality" (3-D worlds). Use "online" only in the context of "online support groups". Complement or substitute product names with broader terms for the class of products (such as "mobile" or "smart phone" instead of "iphone"), especially if the application runs on different platforms.

| | 1 | 2 | 3 | 4 | 5 | |
|---|---|---|---|---|---|---|
| subitem not at all important | ○ | ○ | ◉ | ○ | ○ | essential |

Clear selection

### Does your paper address subitem 1a-i? *

Copy and paste relevant sections from manuscript title (include quotes in quotation marks "like this" to indicate direct quotes from your manuscript), or elaborate on this item by providing additional information not in the ms, or briefly explain why the item is not applicable/relevant for your study

Not applicable - The 3 health information technologies do not fit into the list system and are all a part of management information systems specific to clinical and community healthcare settings.

### 1a-ii) Non-web-based components or important co-interventions in title

Mention non-web-based components or important co-interventions in title, if any (e.g., “with telephone support”).

| | 1 | 2 | 3 | 4 | 5 | |
|---|---|---|---|---|---|---|
| subitem not at all important | ○ | ○ | ◉ | ○ | ○ | essential |

Clear selection

### Does your paper address subitem 1a-ii?

Copy and paste relevant sections from manuscript title (include quotes in quotation marks "like this" to indicate direct quotes from your manuscript), or elaborate on this item by providing additional information not in the ms, or briefly explain why the item is not applicable/relevant for your study

Not applicable - The 3 health information technologies do not fit into the list system and are all a part of management information systems specific to clinical and community healthcare settings.

### 1a-iii) Primary condition or target group in the title

Mention primary condition or target group in the title, if any (e.g., “for children with Type I Diabetes”)
Example: A Web-based and Mobile Intervention with Telephone Support for Children with Type I Diabetes: Randomized Controlled Trial

| | 1 | 2 | 3 | 4 | 5 | |
|---|---|---|---|---|---|---|
| subitem not at all important | ○ | ○ | ○ | ◉ | ○ | essential |

Clear selection

Does your paper address subitem 1a-iii? *

Copy and paste relevant sections from manuscript title (include quotes in quotation marks "like this" to indicate direct quotes from your manuscript), or elaborate on this item by providing additional information not in the ms, or briefly explain why the item is not applicable/relevant for your study

"Parents, Clinicians, and Community Nutritionists"

## 1b) ABSTRACT: Structured summary of trial design, methods, results, and conclusions

NPT extension: Description of experimental treatment, comparator, care providers, centers, and blinding status.

1b-i) Key features/functionalities/components of the intervention and comparator in the METHODS section of the ABSTRACT

Mention key features/functionalities/components of the intervention and comparator in the abstract. If possible, also mention theories and principles used for designing the site. Keep in mind the needs of systematic reviewers and indexers by including important synonyms. (Note: Only report in the abstract what the main paper is reporting. If this information is missing from the main body of text, consider adding it)

| | 1 | 2 | 3 | 4 | 5 | |
|---|---|---|---|---|---|---|
| subitem not at all important | ○ | ○ | ◉ | ○ | ○ | essential |

Clear selection

Does your paper address subitem 1b-i? *

Copy and paste relevant sections from the manuscript abstract (include quotes in quotation marks "like this" to indicate direct quotes from your manuscript), or elaborate on this item by providing additional information not in the ms, or briefly explain why the item is not applicable/relevant for your study

Not applicable - This manuscript only reports on the intervention group of the 2-armed RCT.

### 1b-ii) Level of human involvement in the METHODS section of the ABSTRACT

Clarify the level of human involvement in the abstract, e.g., use phrases like "fully automated" vs. "therapist/nurse/care provider/physician-assisted" (mention number and expertise of providers involved, if any). (Note: Only report in the abstract what the main paper is reporting. If this information is missing from the main body of text, consider adding it)

| | 1 | 2 | 3 | 4 | 5 | |
|---|---|---|---|---|---|---|
| subitem not at all important | ○ | ○ | ○ | ◉ | ○ | essential |

Clear selection

### Does your paper address subitem 1b-ii?

Copy and paste relevant sections from the manuscript abstract (include quotes in quotation marks "like this" to indicate direct quotes from your manuscript), or elaborate on this item by providing additional information not in the ms, or briefly explain why the item is not applicable/relevant for your study

"parents were prompted to respond to the Early Healthy Lifestyles (EHL) self-assessment tool to capture current RP practices" "Care coordination was demonstrated when WIC or PCPs interacted with data and documented RP care provided at the visit"

### 1b-iii) Open vs. closed, web-based (self-assessment) vs. face-to-face assessments in the METHODS section of the ABSTRACT

Mention how participants were recruited (online vs. offline), e.g., from an open access website or from a clinic or a closed online user group (closed usergroup trial), and clarify if this was a purely web-based trial, or there were face-to-face components (as part of the intervention or for assessment). Clearly say if outcomes were self-assessed through questionnaires (as common in web-based trials). Note: In traditional offline trials, an open trial (open-label trial) is a type of clinical trial in which both the researchers and participants know which treatment is being administered. To avoid confusion, use “blinded” or “unblinded” to indicated the level of blinding instead of “open”, as “open” in web-based trials usually refers to “open access” (i.e. participants can self-enrol). (Note: Only report in the abstract what the main paper is reporting. If this information is missing from the main body of text, consider adding it)

| | 1 | 2 | 3 | 4 | 5 | |
|---|---|---|---|---|---|---|
| subitem not at all important | ○ | ○ | ◉ | ○ | ○ | essential |

Clear selection

### Does your paper address subitem 1b-iii?

Copy and paste relevant sections from the manuscript abstract (include quotes in quotation marks "like this" to indicate direct quotes from your manuscript), or elaborate on this item by providing additional information not in the ms, or briefly explain why the item is not applicable/relevant for your study

"newborns receive care from primary-ambulatory-care providers (PCPs) and Women, Infant and Children (WIC) Nutritionists"

### 1b-iv) RESULTS section in abstract must contain use data

Report number of participants enrolled/assessed in each group, the use/uptake of the intervention (e.g., attrition/adherence metrics, use over time, number of logins etc.), in addition to primary/secondary outcomes. (Note: Only report in the abstract what the main paper is reporting. If this information is missing from the main body of text, consider adding it)

| | 1 | 2 | 3 | 4 | 5 | |
|---|---|---|---|---|---|---|
| subitem not at all important | ○ | ○ | ○ | ○ | ◉ | essential |

Clear selection

Does your paper address subitem 1b-iv?

Copy and paste relevant sections from the manuscript abstract (include quotes in quotation marks "like this" to indicate direct quotes from your manuscript), or elaborate on this item by providing additional information not in the ms, or briefly explain why the item is not applicable/relevant for your study

"Dyads (N=131)" - intervention participants only included in manuscript

1b-v) CONCLUSIONS/DISCUSSION in abstract for negative trials

Conclusions/Discussions in abstract for negative trials: Discuss the primary outcome - if the trial is negative (primary outcome not changed), and the intervention was not used, discuss whether negative results are attributable to lack of uptake and discuss reasons. (Note: Only report in the abstract what the main paper is reporting. If this information is missing from the main body of text, consider adding it)

| | 1 | 2 | 3 | 4 | 5 | |
|---|---|---|---|---|---|---|
| subitem not at all important | ○ | ○ | ○ | ◉ | ○ | essential |

Clear selection

Does your paper address subitem 1b-v?

Copy and paste relevant sections from the manuscript abstract (include quotes in quotation marks "like this" to indicate direct quotes from your manuscript), or elaborate on this item by providing additional information not in the ms, or briefly explain why the item is not applicable/relevant for your study

"Although PCPs and WIC shared data, WIC Nutritionists were more likely to use data and document RP care to coordinate care than PCPs. Variability in timing, sequence and frequency of visits underscores the need for flexibility in pragmatic studies"

INTRODUCTION

2a) In INTRODUCTION: Scientific background and explanation of rationale

### 2a-i) Problem and the type of system/solution

Describe the problem and the type of system/solution that is object of the study: intended as stand-alone intervention vs. incorporated in broader health care program? Intended for a particular patient population? Goals of the intervention, e.g., being more cost-effective to other interventions, replace or complement other solutions? (Note: Details about the intervention are provided in “Methods” under 5)

| | 1 | 2 | 3 | 4 | 5 | |
|---|---|---|---|---|---|---|
| subitem not at all important | ○ | ○ | ○ | ○ | ● | essential |

Clear selection

### Does your paper address subitem 2a-i? *

Copy and paste relevant sections from the manuscript (include quotes in quotation marks "like this" to indicate direct quotes from your manuscript), or elaborate on this item by providing additional information not in the ms, or briefly explain why the item is not applicable/relevant for your study

"The objective of this paper is to demonstrate the usability of advanced HIT strategies designed to engage parents in reporting RP practices, integrate data into electronic records to inform and facilitate documentation of provided RP care, and share data between settings to facilitate coordination of care between PCPs and WIC."

### 2a-ii) Scientific background, rationale: What is known about the (type of) system

Scientific background, rationale: What is known about the (type of) system that is the object of the study (be sure to discuss the use of similar systems for other conditions/diagnoses, if appropiate), motivation for the study, i.e. what are the reasons for and what is the context for this specific study, from which stakeholder viewpoint is the study performed, potential impact of findings [2]. Briefly justify the choice of the comparator.

| | 1 | 2 | 3 | 4 | 5 | |
|---|---|---|---|---|---|---|
| subitem not at all important | ○ | ○ | ○ | ○ | ● | essential |

Clear selection

Does your paper address subitem 2a-ii? *

Copy and paste relevant sections from the manuscript (include quotes in quotation marks "like this" to indicate direct quotes from your manuscript), or elaborate on this item by providing additional information not in the ms, or briefly explain why the item is not applicable/relevant for your study

"The integrated and coordinated care model described in this paper applied advanced HIT strategies and represents a novel cross-sector care delivery model to address patient-centered[11,16]. As little is known about the feasibility of cross-sector delivery models, this study addresses a gap by applying and evaluating the usability of advanced HIT capabilities to engage patients, community and clinical providers into a broader patient-centered, integrated and coordinated care process."

## 2b) In INTRODUCTION: Specific objectives or hypotheses

Does your paper address CONSORT subitem 2b? *

Copy and paste relevant sections from the manuscript (include quotes in quotation marks "like this" to indicate direct quotes from your manuscript), or elaborate on this item by providing additional information not in the ms, or briefly explain why the item is not applicable/relevant for your study

"The objective of this paper is to demonstrate the usability of advanced HIT strategies designed to engage parents in reporting RP practices, integrate data into electronic records to inform and facilitate documentation of provided RP care, and share data between settings to facilitate coordination of care between PCPs and WIC."

## METHODS

## 3a) Description of trial design (such as parallel, factorial) including allocation ratio

Does your paper address CONSORT subitem 3a? *

Copy and paste relevant sections from the manuscript (include quotes in quotation marks "like this" to indicate direct quotes from your manuscript), or elaborate on this item by providing additional information not in the ms, or briefly explain why the item is not applicable/relevant for your study

"The objective of this paper is to demonstrate the usability of advanced HIT strategies designed to engage parents in reporting RP practices, integrate data into electronic records to inform and facilitate documentation of provided RP care, and share data between settings to facilitate coordination of care between PCPs and WIC."

**3b) Important changes to methods after trial commencement (such as eligibility criteria), with reasons**

Does your paper address CONSORT subitem 3b? *

Copy and paste relevant sections from the manuscript (include quotes in quotation marks "like this" to indicate direct quotes from your manuscript), or elaborate on this item by providing additional information not in the ms, or briefly explain why the item is not applicable/relevant for your study

Not applicable

3b-i) Bug fixes, Downtimes, Content Changes

Bug fixes, Downtimes, Content Changes: ehealth systems are often dynamic systems. A description of changes to methods therefore also includes important changes made on the intervention or comparator during the trial (e.g., major bug fixes or changes in the functionality or content) (5-iii) and other “unexpected events” that may have influenced study design such as staff changes, system failures/downtimes, etc. [2].

| | 1 | 2 | 3 | 4 | 5 | |
|---|---|---|---|---|---|---|
| subitem not at all important | ○ | ○ | ○ | ◉ | ○ | essential |

Clear selection

Does your paper address subitem 3b-i?

Copy and paste relevant sections from the manuscript (include quotes in quotation marks "like this" to indicate direct quotes from your manuscript), or elaborate on this item by providing additional information not in the ms, or briefly explain why the item is not applicable/relevant for your study

"Due to the secure and study staff-dependent data sharing processes, the receiving provider was able to view the shared data within 2 business days if sent by PCP and 1 business day if sent by WIC."

## 4a) Eligibility criteria for participants

Does your paper address CONSORT subitem 4a? *

Copy and paste relevant sections from the manuscript (include quotes in quotation marks "like this" to indicate direct quotes from your manuscript), or elaborate on this item by providing additional information not in the ms, or briefly explain why the item is not applicable/relevant for your study

"Mother-infant dyads were either recruited in-person in labor and delivery unit or on-the-phone after the first WCV. Eligible dyads met the following inclusion criteria: full-term (≥37 weeks gestation), singleton newborn, English-speaking mother between 18 and 55 years old, intended to receive well-child care at a participating pediatric clinic, and eligible to enroll or enrolled in WIC."

4a-i) Computer / Internet literacy

Computer / Internet literacy is often an implicit “de facto” eligibility criterion - this should be explicitly clarified.

| | 1 | 2 | 3 | 4 | 5 | |
|---|---|---|---|---|---|---|
| subitem not at all important | ○ | ○ | ◉ | ○ | ○ | essential |

Clear selection

### Does your paper address subitem 4a-i?

Copy and paste relevant sections from the manuscript (include quotes in quotation marks "like this" to indicate direct quotes from your manuscript), or elaborate on this item by providing additional information not in the ms, or briefly explain why the item is not applicable/relevant for your study

"Prior to the start of the intervention, participating PCPs were trained to evaluate the EHL data, deliver tailored RP messages that aligned with parent learning needs at WCV, and document provided RP care in the study-specific table in the WVC progress note, herein referred to as the PCP System." "WIC Nutritionist received training on this process, use of EHL data, and delivery of RP care messages prior to the start of the intervention. A booster training session was provided in the 14th month of the intervention."

### 4a-ii) Open vs. closed, web-based vs. face-to-face assessments:

Open vs. closed, web-based vs. face-to-face assessments: Mention how participants were recruited (online vs. offline), e.g., from an open access website or from a clinic, and clarify if this was a purely web-based trial, or there were face-to-face components (as part of the intervention or for assessment), i.e., to what degree got the study team to know the participant. In online-only trials, clarify if participants were quasi-anonymous and whether having multiple identities was possible or whether technical or logistical measures (e.g., cookies, email confirmation, phone calls) were used to detect/prevent these.

| | 1 | 2 | 3 | 4 | 5 | |
|---|---|---|---|---|---|---|
| subitem not at all important | ○ | ○ | ○ | ◉ | ○ | essential |

Clear selection

### Does your paper address subitem 4a-ii? *

Copy and paste relevant sections from the manuscript (include quotes in quotation marks "like this" to indicate direct quotes from your manuscript), or elaborate on this item by providing additional information not in the ms, or briefly explain why the item is not applicable/relevant for your study

"Mother-infant dyads were either recruited in-person in labor and delivery unit or on-the-phone after the first WCV. "

## 4a-iii) Information giving during recruitment

Information given during recruitment. Specify how participants were briefed for recruitment and in the informed consent procedures (e.g., publish the informed consent documentation as appendix, see also item X26), as this information may have an effect on user self-selection, user expectation and may also bias results.

| | 1 | 2 | 3 | 4 | 5 | |
|---|---|---|---|---|---|---|
| subitem not at all important | ○ | ◉ | ○ | ○ | ○ | essential |

Clear selection

## Does your paper address subitem 4a-iii?

Copy and paste relevant sections from the manuscript (include quotes in quotation marks "like this" to indicate direct quotes from your manuscript), or elaborate on this item by providing additional information not in the ms, or briefly explain why the item is not applicable/relevant for your study

"The integrated and care coordination delivery model was constructed for the WEE Baby Care Study, that has been described elsewhere[20]. " "Participants provided written informed consent."

## 4b) Settings and locations where the data were collected

## Does your paper address CONSORT subitem 4b? *

Copy and paste relevant sections from the manuscript (include quotes in quotation marks "like this" to indicate direct quotes from your manuscript), or elaborate on this item by providing additional information not in the ms, or briefly explain why the item is not applicable/relevant for your study

"The parent was prompted by electronic messaging to complete the EHL in the patient portal prior to the WCV. Mother’s proxy access to child’s portal was ensured at study enrollment. If the parent did not complete the tool, clinic staff encouraged the parent to complete the EHL in the waiting room on a tablet. "

### 4b-i) Report if outcomes were (self-)assessed through online questionnaires

Clearly report if outcomes were (self-)assessed through online questionnaires (as common in web-based trials) or otherwise.

| | 1 | 2 | 3 | 4 | 5 | |
|---|---|---|---|---|---|---|
| subitem not at all important | ○ | ○ | ◉ | ○ | ○ | essential |

Clear selection

### Does your paper address subitem 4b-i? *

Copy and paste relevant sections from the manuscript (include quotes in quotation marks "like this" to indicate direct quotes from your manuscript), or elaborate on this item by providing additional information not in the ms, or briefly explain why the item is not applicable/relevant for your study

Not applicable - No outcomes in the manuscript were self-assessed.

### 4b-ii) Report how institutional affiliations are displayed

Report how institutional affiliations are displayed to potential participants [on ehealth media], as affiliations with prestigious hospitals or universities may affect volunteer rates, use, and reactions with regards to an intervention.(Not a required item – describe only if this may bias results)

| | 1 | 2 | 3 | 4 | 5 | |
|---|---|---|---|---|---|---|
| subitem not at all important | ○ | ◉ | ○ | ○ | ○ | essential |

Clear selection

### Does your paper address subitem 4b-ii?

Copy and paste relevant sections from the manuscript (include quotes in quotation marks "like this" to indicate direct quotes from your manuscript), or elaborate on this item by providing additional information not in the ms, or briefly explain why the item is not applicable/relevant for your study

Not applicable - Applications were integrated into their standard well-child visits and WIC visits.

## 5) The interventions for each group with sufficient details to allow replication, including how and when they were actually administered

### 5-i) Mention names, credential, affiliations of the developers, sponsors, and owners

Mention names, credential, affiliations of the developers, sponsors, and owners [6] (if authors/evaluators are owners or developer of the software, this needs to be declared in a “Conflict of interest” section or mentioned elsewhere in the manuscript).

| | 1 | 2 | 3 | 4 | 5 | |
|---|---|---|---|---|---|---|
| subitem not at all important | ○ | ○ | ● | ○ | ○ | essential |

Clear selection

### Does your paper address subitem 5-i?

Copy and paste relevant sections from the manuscript (include quotes in quotation marks "like this" to indicate direct quotes from your manuscript), or elaborate on this item by providing additional information not in the ms, or briefly explain why the item is not applicable/relevant for your study

"Development of the PCP System was guided, approved, and integrated into the EHR by Geisinger’s Health Information Technology Team." "An application developer at the Pennsylvania Department of Health developed and implemented the WIC System as an external cloud system, as opposed to integrated system, as state-wide plans were underway to replace the QuickWIC system."

## 5-ii) Describe the history/development process

Describe the history/development process of the application and previous formative evaluations (e.g., focus groups, usability testing), as these will have an impact on adoption/use rates and help with interpreting results.

| | 1 | 2 | 3 | 4 | 5 | |
|---|---|---|---|---|---|---|
| subitem not at all important | ○ | ○ | ◉ | ○ | ○ | essential |

Clear selection

## Does your paper address subitem 5-ii?

Copy and paste relevant sections from the manuscript (include quotes in quotation marks "like this" to indicate direct quotes from your manuscript), or elaborate on this item by providing additional information not in the ms, or briefly explain why the item is not applicable/relevant for your study

Not applicable - reported in prior, published manuscript

## 5-iii) Revisions and updating

Revisions and updating. Clearly mention the date and/or version number of the application/intervention (and comparator, if applicable) evaluated, or describe whether the intervention underwent major changes during the evaluation process, or whether the development and/or content was “frozen” during the trial. Describe dynamic components such as news feeds or changing content which may have an impact on the replicability of the intervention (for unexpected events see item 3b).

| | 1 | 2 | 3 | 4 | 5 | |
|---|---|---|---|---|---|---|
| subitem not at all important | ○ | ○ | ◉ | ○ | ○ | essential |

Clear selection

### Does your paper address subitem 5-iii?

Copy and paste relevant sections from the manuscript (include quotes in quotation marks "like this" to indicate direct quotes from your manuscript), or elaborate on this item by providing additional information not in the ms, or briefly explain why the item is not applicable/relevant for your study

Not applicable - no major revisions occurred and thus are not reported

### 5-iv) Quality assurance methods

Provide information on quality assurance methods to ensure accuracy and quality of information provided [1], if applicable.

| | 1 | 2 | 3 | 4 | 5 | |
|---|---|---|---|---|---|---|
| subitem not at all important | ○ | ○ | ○ | ◉ | ○ | essential |

Clear selection

### Does your paper address subitem 5-iv?

Copy and paste relevant sections from the manuscript (include quotes in quotation marks "like this" to indicate direct quotes from your manuscript), or elaborate on this item by providing additional information not in the ms, or briefly explain why the item is not applicable/relevant for your study

"The coding process was operationally detailed in a study-team-developed coding manual that was extensively reviewed multiple times by five study team members (SMRK, HAH, MM, JSS, LBD), and applied to a comprehensive, single data set. The data set included data from the PCP System and the WIC System along with other data elements listed in Table 1 to identify data sharing, care coordination opportunities, and care coordination outcomes. Two postdoctoral-level study team members (SMRK, HAH) coded the data independently, discrepancies were discussed and resolved using the coding manual, and both team members recoded the discrepant observations. After the first round of coding, 86% of coded observations agreed between the two coders demonstrating adequate understanding of the coding process. This process continued until the two sets of coded data completely matched. "

## 5-v) Ensure replicability by publishing the source code, and/or providing screenshots/screen-capture video, and/or providing flowcharts of the algorithms used

Ensure replicability by publishing the source code, and/or providing screenshots/screen-capture video, and/or providing flowcharts of the algorithms used. Replicability (i.e., other researchers should in principle be able to replicate the study) is a hallmark of scientific reporting.

| | 1 | 2 | 3 | 4 | 5 | |
|---|---|---|---|---|---|---|
| subitem not at all important | ○ | ○ | ○ | ◉ | ○ | essential |

Clear selection

## Does your paper address subitem 5-v?

Copy and paste relevant sections from the manuscript (include quotes in quotation marks "like this" to indicate direct quotes from your manuscript), or elaborate on this item by providing additional information not in the ms, or briefly explain why the item is not applicable/relevant for your study

See Figure 2 and 3

## 5-vi) Digital preservation

Digital preservation: Provide the URL of the application, but as the intervention is likely to change or disappear over the course of the years; also make sure the intervention is archived (Internet Archive, webcitation.org, and/or publishing the source code or screenshots/videos alongside the article). As pages behind login screens cannot be archived, consider creating demo pages which are accessible without login.

| | 1 | 2 | 3 | 4 | 5 | |
|---|---|---|---|---|---|---|
| subitem not at all important | ○ | ○ | ◉ | ○ | ○ | essential |

Clear selection

## Does your paper address subitem 5-vi?

Copy and paste relevant sections from the manuscript (include quotes in quotation marks "like this" to indicate direct quotes from your manuscript), or elaborate on this item by providing additional information not in the ms, or briefly explain why the item is not applicable/relevant for your study

Not applicable - corresponding author can be contacted for screenshots

## 5-vii) Access

Access: Describe how participants accessed the application, in what setting/context, if they had to pay (or were paid) or not, whether they had to be a member of specific group. If known, describe how participants obtained "access to the platform and Internet" [1]. To ensure access for editors/reviewers/readers, consider to provide a "backdoor" login account or demo mode for reviewers/readers to explore the application (also important for archiving purposes, see vi).

| | 1 | 2 | 3 | 4 | 5 | |
|---|---|---|---|---|---|---|
| subitem not at all important | ○ | ○ | ○ | ◉ | ○ | essential |

Clear selection

## Does your paper address subitem 5-vii? *

Copy and paste relevant sections from the manuscript (include quotes in quotation marks "like this" to indicate direct quotes from your manuscript), or elaborate on this item by providing additional information not in the ms, or briefly explain why the item is not applicable/relevant for your study

"Separate logins were required for each system. " "Uniquely, the WIC System required the Nutritionist to sign off on the record after the visit was completed to initiate data sharing."

### 5-viii) Mode of delivery, features/functionalities/components of the intervention and comparator, and the theoretical framework

Describe mode of delivery, features/functionalities/components of the intervention and comparator, and the theoretical framework [6] used to design them (instructional strategy [1], behaviour change techniques, persuasive features, etc., see e.g., [7, 8] for terminology). This includes an in-depth description of the content (including where it is coming from and who developed it) [1]," whether [and how] it is tailored to individual circumstances and allows users to track their progress and receive feedback" [6]. This also includes a description of communication delivery channels and – if computer-mediated communication is a component – whether communication was synchronous or asynchronous [6]. It also includes information on presentation strategies [1], including page design principles, average amount of text on pages, presence of hyperlinks to other resources, etc. [1].

| | 1 | 2 | 3 | 4 | 5 | |
|---|---|---|---|---|---|---|
| subitem not at all important | ○ | ○ | ○ | ◉ | ○ | essential |

Clear selection

### Does your paper address subitem 5-viii? *

Copy and paste relevant sections from the manuscript (include quotes in quotation marks "like this" to indicate direct quotes from your manuscript), or elaborate on this item by providing additional information not in the ms, or briefly explain why the item is not applicable/relevant for your study

Not applicable - only intervention arm of the 2-arm RCT is reported on.

### 5-ix) Describe use parameters

Describe use parameters (e.g., intended "doses" and optimal timing for use). Clarify what instructions or recommendations were given to the user, e.g., regarding timing, frequency, heaviness of use, if any, or was the intervention used ad libitum.

| | 1 | 2 | 3 | 4 | 5 | |
|---|---|---|---|---|---|---|
| subitem not at all important | ○ | ○ | ◉ | ○ | ○ | essential |

Clear selection

### Does your paper address subitem 5-ix?

Copy and paste relevant sections from the manuscript (include quotes in quotation marks "like this" to indicate direct quotes from your manuscript), or elaborate on this item by providing additional information not in the ms, or briefly explain why the item is not applicable/relevant for your study

"Of the expected 4 WCV and 2 WIC visits per dyad to be experienced in the observation period, 45.8% of dyads attended 4 or more WCVs and 77.9% attended 2 or more WIC visits."

### 5-x) Clarify the level of human involvement

Clarify the level of human involvement (care providers or health professionals, also technical assistance) in the e-intervention or as co-intervention (detail number and expertise of professionals involved, if any, as well as “type of assistance offered, the timing and frequency of the support, how it is initiated, and the medium by which the assistance is delivered”. It may be necessary to distinguish between the level of human involvement required for the trial, and the level of human involvement required for a routine application outside of a RCT setting (discuss under item 21 – generalizability).

| | 1 | 2 | 3 | 4 | 5 | |
|---|---|---|---|---|---|---|
| subitem not at all important | ○ | ○ | ○ | ○ | ◉ | essential |

Clear selection

Does your paper address subitem 5-x?

Copy and paste relevant sections from the manuscript (include quotes in quotation marks "like this" to indicate direct quotes from your manuscript), or elaborate on this item by providing additional information not in the ms, or briefly explain why the item is not applicable/relevant for your study

"Mother-infant dyads randomized to the intervention group completed the Early Healthy Lifestyles (EHL) risk assessment tool prior to each WCV. " "The parent was prompted by electronic messaging to complete the EHL in the patient portal prior to the WCV. Mother’s proxy access to child’s portal was ensured at study enrollment. If the parent did not complete the tool, clinic staff encouraged the parent to complete the EHL in the waiting room on a tablet." "Within the WCV progress note, PCPs could view and use the EHL data to inform and document patient-centered RP care. Prior to the start of the intervention, participating PCPs were trained to evaluate the EHL data, deliver tailored RP messages that aligned with parent learning needs at WCV, and document provided RP care in the study-specific table in the WVC progress note, herein referred to as the PCP System. Age appropriate, preventive messages were informed by the INSIGHT study and AAP Healthy Active Living for Families (HALF) curriculum[17,22,23]. PCPs used progress note functions to facilitate documentation of RP care provided during the WCV. Additionally, PCPs could add an 80-character free-text comment to send to the WIC Nutritionist, perhaps to direct attention to a high priority issue." "The WIC System extracted education codes from QuickWIC. Following the visit with the trained WIC Nutritionist, encounter data of interest (Table 1) were extracted from the WIC System and securely shared from WIC to the PCP, thus providing a communication feedback loop to coordinate care at subsequent visits. Uniquely, the WIC System required the Nutritionist to sign off on the record after the visit was completed to initiate data sharing. WIC Nutritionist received training on this process, use of EHL data, and delivery of RP care messages prior to the start of the intervention."

### 5-xi) Report any prompts/reminders used

Report any prompts/reminders used: Clarify if there were prompts (letters, emails, phone calls, SMS) to use the application, what triggered them, frequency etc. It may be necessary to distinguish between the level of prompts/reminders required for the trial, and the level of prompts/reminders for a routine application outside of a RCT setting (discuss under item 21 – generalizability).

| | 1 | 2 | 3 | 4 | 5 | |
|---|---|---|---|---|---|---|
| subitem not at all important | ○ | ○ | ◉ | ○ | ○ | essential |

Clear selection

### Does your paper address subitem 5-xi? *

Copy and paste relevant sections from the manuscript (include quotes in quotation marks "like this" to indicate direct quotes from your manuscript), or elaborate on this item by providing additional information not in the ms, or briefly explain why the item is not applicable/relevant for your study

"The parent was prompted by electronic messaging to complete the EHL in the patient portal prior to the WCV. Mother’s proxy access to child’s portal was ensured at study enrollment. If the parent did not complete the tool, clinic staff encouraged the parent to complete the EHL in the waiting room on a tablet. "

### 5-xii) Describe any co-interventions (incl. training/support)

Describe any co-interventions (incl. training/support): Clearly state any interventions that are provided in addition to the targeted eHealth intervention, as ehealth intervention may not be designed as stand-alone intervention. This includes training sessions and support [1]. It may be necessary to distinguish between the level of training required for the trial, and the level of training for a routine application outside of a RCT setting (discuss under item 21 – generalizability.

| | 1 | 2 | 3 | 4 | 5 | |
|---|---|---|---|---|---|---|
| subitem not at all important | ○ | ○ | ◉ | ○ | ○ | essential |

Clear selection

Does your paper address subitem 5-xii? *

Copy and paste relevant sections from the manuscript (include quotes in quotation marks "like this" to indicate direct quotes from your manuscript), or elaborate on this item by providing additional information not in the ms, or briefly explain why the item is not applicable/relevant for your study

"Prior to the start of the intervention, participating PCPs were trained to evaluate the EHL data, deliver tailored RP messages that aligned with parent learning needs at WCV, and document provided RP care in the study-specific table in the WVC progress note, herein referred to as the PCP System. " "WIC Nutritionist received training on this process, use of EHL data, and delivery of RP care messages prior to the start of the intervention. A booster training session was provided in the 14th month of the intervention. "

6a) Completely defined pre-specified primary and secondary outcome measures, including how and when they were assessed

Does your paper address CONSORT subitem 6a? *

Copy and paste relevant sections from the manuscript (include quotes in quotation marks "like this" to indicate direct quotes from your manuscript), or elaborate on this item by providing additional information not in the ms, or briefly explain why the item is not applicable/relevant for your study

This manuscript does not report on the primary outcomes of the RCT. "The outcomes of interest included the utilization of the advanced HIT strategies and the feasibility of data sharing and care coordination occurring in two directions, PCP to WIC and WIC to PCP. Data Sharing is the electronic and systematic push of study-related data through the electronic data warehouse (distinct from EHR and PCP System) managed by the research team in the clinical setting. Data Sharing does not require the PCP or WIC Nutritionist in the receiving setting to interact with the data of interest. A Care Coordination Opportunity is nested within Data Sharing and is an opportunity for a PCP or WIC Nutritionist to view data for the most recent visit from the sending setting. Due to the secure and study staff-dependent data sharing processes, the receiving provider was able to view the shared data within 2 business days if sent by PCP and 1 business day if sent by WIC. Eligible WCVs occurred after the dyad was enrolled into the study and occurred up to 6 months of infant's age at which time the clinical study team turned off the PCP System. Eligible WIC visits occurred after the dyad was enrolled in the study but before disabling the PCP System.

Care Coordination is nested within Care Coordination Opportunity and requires a PCP or WIC Nutritionist in the receiving setting to be able to access the sent data AND document provided RP care. The study team could not directly verify that the receiving setting interacted with the sent data as accessing or viewing the data was not captured with a discrete data point; thus, the documentation of RP care serves as proxy evidence that the receiving provider used the shared data to coordinate care. Preventive RP care included educational messages that addressed parent learning needs as self-assessed by parents using the EHL tool. Preventive RP care was documented by PCPs and WIC Nutritionists by selecting RP topics and/or writing a free-text comment.
"

6a-i) Online questionnaires: describe if they were validated for online use and apply CHERRIES items to describe how the questionnaires were designed/deployed

If outcomes were obtained through online questionnaires, describe if they were validated for online use and apply CHERRIES items to describe how the questionnaires were designed/deployed [9].

| | 1 | 2 | 3 | 4 | 5 | |
|---|---|---|---|---|---|---|
| subitem not at all important | ○ | ○ | ○ | ◉ | ○ | essential |

Clear selection

Does your paper address subitem 6a-i?

Copy and paste relevant sections from manuscript text

Not applicable - response to the Early Healthy Living tool are not essential nor reported in this manuscript.

6a-ii) Describe whether and how “use” (including intensity of use/dosage) was defined/measured/monitored

Describe whether and how “use” (including intensity of use/dosage) was defined/measured/monitored (logins, logfile analysis, etc.). Use/adoption metrics are important process outcomes that should be reported in any ehealth trial.

| | 1 | 2 | 3 | 4 | 5 | |
|---|---|---|---|---|---|---|
| subitem not at all important | ○ | ○ | ○ | ○ | ◉ | essential |

Clear selection

Does your paper address subitem 6a-ii?

Copy and paste relevant sections from manuscript text

Data Sharing is the electronic and systematic push of study-related data through the electronic data warehouse (distinct from EHR and PCP System) managed by the research team in the clinical setting. Data Sharing does not require the PCP or WIC Nutritionist in the receiving setting to interact with the data of interest (Table 1).
A Care Coordination Opportunity is nested within Data Sharing and is an opportunity for a PCP or WIC Nutritionist to view data for the most recent visit from the sending setting. Due to the secure and study staff-dependent data sharing processes, the receiving provider was able to view the shared data within 2 business days if sent by PCP and 1 business day if sent by WIC. Eligible WCVs occurred after the dyad was enrolled into the study and occurred up to 6 months of infant's age at which time the clinical study team turned off the PCP System. Eligible WIC visits occurred after the dyad was enrolled in the study but before disabling the PCP System.
Care Coordination is nested within Care Coordination Opportunity and requires a PCP or WIC Nutritionist in the receiving setting to be able to access the sent data AND document provided RP care. The study team could not directly verify that the receiving setting interacted with the sent data as accessing or viewing the data was not captured with a discrete data point; thus, the documentation of RP care serves as proxy evidence that the receiving provider used the shared data to coordinate care. Preventive RP care included educational messages that addressed parent learning needs as self-assessed by parents using the EHL tool. Preventive RP care was documented by PCPs and WIC Nutritionists by selecting RP topics and/or writing a free-text comment.

6a-iii) Describe whether, how, and when qualitative feedback from participants was obtained

Describe whether, how, and when qualitative feedback from participants was obtained (e.g., through emails, feedback forms, interviews, focus groups).

| | 1 | 2 | 3 | 4 | 5 | |
|---|---|---|---|---|---|---|
| subitem not at all important | ○ | ○ | ○ | ◉ | ○ | essential |

Clear selection

Does your paper address subitem 6a-iii?

Copy and paste relevant sections from manuscript text

Not applicable - qualitative results from formative research have been previously reported and published.

6b) Any changes to trial outcomes after the trial commenced, with reasons

Does your paper address CONSORT subitem 6b? *

Copy and paste relevant sections from the manuscript (include quotes in quotation marks "like this" to indicate direct quotes from your manuscript), or elaborate on this item by providing additional information not in the ms, or briefly explain why the item is not applicable/relevant for your study

Not applicable - Outcomes reported here were exploratory in the feasibility study.

7a) How sample size was determined

NPT: When applicable, details of whether and how the clustering by care provides or centers was addressed

### 7a-i) Describe whether and how expected attrition was taken into account when calculating the sample size

Describe whether and how expected attrition was taken into account when calculating the sample size.

| | 1 | 2 | 3 | 4 | 5 | |
|---|---|---|---|---|---|---|
| subitem not at all important | ○ | ○ | ○ | ◉ | ○ | essential |

Clear selection

### Does your paper address subitem 7a-i?

Copy and paste relevant sections from manuscript title (include quotes in quotation marks "like this" to indicate direct quotes from your manuscript), or elaborate on this item by providing additional information not in the ms, or briefly explain why the item is not applicable/relevant for your study

Not applicable - Sample size determination has been previously reported.

### 7b) When applicable, explanation of any interim analyses and stopping guidelines

### Does your paper address CONSORT subitem 7b? *

Copy and paste relevant sections from the manuscript (include quotes in quotation marks "like this" to indicate direct quotes from your manuscript), or elaborate on this item by providing additional information not in the ms, or briefly explain why the item is not applicable/relevant for your study

Not applicable - interim analyses were not conducted

### 8a) Method used to generate the random allocation sequence

NPT: When applicable, how care providers were allocated to each trial group

Does your paper address CONSORT subitem 8a? *

Copy and paste relevant sections from the manuscript (include quotes in quotation marks "like this" to indicate direct quotes from your manuscript), or elaborate on this item by providing additional information not in the ms, or briefly explain why the item is not applicable/relevant for your study

Not applicable - randomization procedures have been previously reported

**8b) Type of randomisation; details of any restriction (such as blocking and block size)**

Does your paper address CONSORT subitem 8b? *

Copy and paste relevant sections from the manuscript (include quotes in quotation marks "like this" to indicate direct quotes from your manuscript), or elaborate on this item by providing additional information not in the ms, or briefly explain why the item is not applicable/relevant for your study

Not applicable - randomization procedures have been previously reported

**9) Mechanism used to implement the random allocation sequence (such as sequentially numbered containers), describing any steps taken to conceal the sequence until interventions were assigned**

Does your paper address CONSORT subitem 9? *

Copy and paste relevant sections from the manuscript (include quotes in quotation marks "like this" to indicate direct quotes from your manuscript), or elaborate on this item by providing additional information not in the ms, or briefly explain why the item is not applicable/relevant for your study

Not applicable - randomization procedures have been previously reported

**10) Who generated the random allocation sequence, who enrolled participants, and who assigned participants to interventions**

Does your paper address CONSORT subitem 10? *

Copy and paste relevant sections from the manuscript (include quotes in quotation marks "like this" to indicate direct quotes from your manuscript), or elaborate on this item by providing additional information not in the ms, or briefly explain why the item is not applicable/relevant for your study

Not applicable - randomization procedures have been previously reported

### 11a) If done, who was blinded after assignment to interventions (for example, participants, care providers, those assessing outcomes) and how

NPT: Whether or not administering co-interventions were blinded to group assignment

11a-i) Specify who was blinded, and who wasn't

Specify who was blinded, and who wasn't. Usually, in web-based trials it is not possible to blind the participants [1, 3] (this should be clearly acknowledged), but it may be possible to blind outcome assessors, those doing data analysis or those administering co-interventions (if any).

| | 1 | 2 | 3 | 4 | 5 | |
|---|---|---|---|---|---|---|
| subitem not at all important | ○ | ○ | ◉ | ○ | ○ | essential |

Clear selection

Does your paper address subitem 11a-i? *

Copy and paste relevant sections from the manuscript (include quotes in quotation marks "like this" to indicate direct quotes from your manuscript), or elaborate on this item by providing additional information not in the ms, or briefly explain why the item is not applicable/relevant for your study

Not applicable - randomization procedures have been previously reported

## 11a-ii) Discuss e.g., whether participants knew which intervention was the “intervention of interest” and which one was the “comparator”

Informed consent procedures (4a-ii) can create biases and certain expectations - discuss e.g., whether participants knew which intervention was the “intervention of interest” and which one was the “comparator”.

| | 1 | 2 | 3 | 4 | 5 | |
|---|---|---|---|---|---|---|
| subitem not at all important | ○ | ○ | ● | ○ | ○ | essential |

Clear selection

## Does your paper address subitem 11a-ii?

Copy and paste relevant sections from the manuscript (include quotes in quotation marks "like this" to indicate direct quotes from your manuscript), or elaborate on this item by providing additional information not in the ms, or briefly explain why the item is not applicable/relevant for your study

Not applicable - this manuscript only reports on the intervention participants.

## 11b) If relevant, description of the similarity of interventions

(this item is usually not relevant for ehealth trials as it refers to similarity of a placebo or sham intervention to a active medication/intervention)

## Does your paper address CONSORT subitem 11b? *

Copy and paste relevant sections from the manuscript (include quotes in quotation marks "like this" to indicate direct quotes from your manuscript), or elaborate on this item by providing additional information not in the ms, or briefly explain why the item is not applicable/relevant for your study

Not applicable - the intervention arm of a 2-arm RCT is the focus of this study.

## 12a) Statistical methods used to compare groups for primary and secondary outcomes

NPT: When applicable, details of whether and how the clustering by care providers or centers was addressed

Does your paper address CONSORT subitem 12a? *

Copy and paste relevant sections from the manuscript (include quotes in quotation marks "like this" to indicate direct quotes from your manuscript), or elaborate on this item by providing additional information not in the ms, or briefly explain why the item is not applicable/relevant for your study

Not applicable - only descriptive statistics are produced for the intervention arm in this manuscript.

12a-i) Imputation techniques to deal with attrition / missing values

Imputation techniques to deal with attrition / missing values: Not all participants will use the intervention/comparator as intended and attrition is typically high in ehealth trials. Specify how participants who did not use the application or dropped out from the trial were treated in the statistical analysis (a complete case analysis is strongly discouraged, and simple imputation techniques such as LOCF may also be problematic [4]).

| | 1 | 2 | 3 | 4 | 5 | |
|---|---|---|---|---|---|---|
| subitem not at all important | ○ | ○ | ○ | ◉ | ○ | essential |

Clear selection

Does your paper address subitem 12a-i? *

Copy and paste relevant sections from the manuscript (include quotes in quotation marks "like this" to indicate direct quotes from your manuscript), or elaborate on this item by providing additional information not in the ms, or briefly explain why the item is not applicable/relevant for your study

Not applicable - no imputation techniques were applied to this data.

12b) Methods for additional analyses, such as subgroup analyses and adjusted analyses

Does your paper address CONSORT subitem 12b? *

Copy and paste relevant sections from the manuscript (include quotes in quotation marks "like this" to indicate direct quotes from your manuscript), or elaborate on this item by providing additional information not in the ms, or briefly explain why the item is not applicable/relevant for your study

Not applicable - analyses of subgroups of the intervention are not a component of this manuscript

## X26) REB/IRB Approval and Ethical Considerations [recommended as subheading under "Methods"] (not a CONSORT item)

X26-i) Comment on ethics committee approval

| | 1 | 2 | 3 | 4 | 5 | |
|---|---|---|---|---|---|---|
| subitem not at all important | ○ | ○ | ○ | ○ | ◉ | essential |

Clear selection

Does your paper address subitem X26-i?

Copy and paste relevant sections from the manuscript (include quotes in quotation marks "like this" to indicate direct quotes from your manuscript), or elaborate on this item by providing additional information not in the ms, or briefly explain why the item is not applicable/relevant for your study

"All study procedures were approved by the Institutional Review Boards of The Pennsylvania State University and Geisinger."

### x26-ii) Outline informed consent procedures

Outline informed consent procedures e.g., if consent was obtained offline or online (how? Checkbox, etc.?), and what information was provided (see 4a-ii). See [6] for some items to be included in informed consent documents.

| | 1 | 2 | 3 | 4 | 5 | |
|---|---|---|---|---|---|---|
| subitem not at all important | ○ | ○ | ○ | ○ | ● | essential |

Clear selection

### Does your paper address subitem X26-ii?

Copy and paste relevant sections from the manuscript (include quotes in quotation marks "like this" to indicate direct quotes from your manuscript), or elaborate on this item by providing additional information not in the ms, or briefly explain why the item is not applicable/relevant for your study

"The integrated and care coordination delivery model was constructed for the WEE Baby Care Study, that has been described elsewhere[20]." "Participants provided written informed consent. All study procedures were approved by the Institutional Review Boards of The Pennsylvania State University and Geisinger."

### X26-iii) Safety and security procedures

Safety and security procedures, incl. privacy considerations, and any steps taken to reduce the likelihood or detection of harm (e.g., education and training, availability of a hotline)

| | 1 | 2 | 3 | 4 | 5 | |
|---|---|---|---|---|---|---|
| subitem not at all important | ○ | ○ | ○ | ● | ○ | essential |

Clear selection

Does your paper address subitem X26-iii?

Copy and paste relevant sections from the manuscript (include quotes in quotation marks "like this" to indicate direct quotes from your manuscript), or elaborate on this item by providing additional information not in the ms, or briefly explain why the item is not applicable/relevant for your study

"Prior to the start of the intervention, participating PCPs were trained to evaluate the EHL data, deliver tailored RP messages that aligned with parent learning needs at WCV, and document provided RP care in the study-specific table in the WVC progress note, herein referred to as the PCP System." "WIC Nutritionist received training on this process, use of EHL data, and delivery of RP care messages prior to the start of the intervention. A booster training session was provided in the 14th month of the intervention."

## RESULTS

### 13a) For each group, the numbers of participants who were randomly assigned, received intended treatment, and were analysed for the primary outcome

NPT: The number of care providers or centers performing the intervention in each group and the number of patients treated by each care provider in each center

Does your paper address CONSORT subitem 13a? *

Copy and paste relevant sections from the manuscript (include quotes in quotation marks "like this" to indicate direct quotes from your manuscript), or elaborate on this item by providing additional information not in the ms, or briefly explain why the item is not applicable/relevant for your study

"The 131 intervention mother-infant dyads" - the intervention group is the focus of this manuscript and thus the control group is not described.

### 13b) For each group, losses and exclusions after randomisation, together with reasons

Does your paper address CONSORT subitem 13b? (NOTE: Preferably, this is shown in a CONSORT flow diagram) *

Copy and paste relevant sections from the manuscript (include quotes in quotation marks "like this" to indicate direct quotes from your manuscript), or elaborate on this item by providing additional information not in the ms, or briefly explain why the item is not applicable/relevant for your study

Not applicable - the CONSORT flow diagram is not applicable to this paper as it only includes data from the intervention arm of the 2-arm RCT and the CONSORT flow diagram will be included in the publication of the primary outcomes of the RCT.

13b-i) Attrition diagram

Strongly recommended: An attrition diagram (e.g., proportion of participants still logging in or using the intervention/comparator in each group plotted over time, similar to a survival curve) or other figures or tables demonstrating usage/dose/engagement.

| | 1 | 2 | 3 | 4 | 5 | |
|---|---|---|---|---|---|---|
| subitem not at all important | ○ | ○ | ● | ○ | ○ | essential |

Clear selection

Does your paper address subitem 13b-i?

Copy and paste relevant sections from the manuscript or cite the figure number if applicable (include quotes in quotation marks "like this" to indicate direct quotes from your manuscript), or elaborate on this item by providing additional information not in the ms, or briefly explain why the item is not applicable/relevant for your study

Not applicable - the CONSORT flow diagram is not applicable to this paper as it only includes data from the intervention arm of the 2-arm RCT and the CONSORT flow diagram will be included in the publication of the primary outcomes of the RCT.

14a) Dates defining the periods of recruitment and follow-up

Does your paper address CONSORT subitem 14a? *

Copy and paste relevant sections from the manuscript (include quotes in quotation marks "like this" to indicate direct quotes from your manuscript), or elaborate on this item by providing additional information not in the ms, or briefly explain why the item is not applicable/relevant for your study

"From July 2016 to May 2018"

14a-i) Indicate if critical “secular events” fell into the study period

Indicate if critical “secular events” fell into the study period, e.g., significant changes in Internet resources available or “changes in computer hardware or Internet delivery resources”

| | 1 | 2 | 3 | 4 | 5 | |
|---|---|---|---|---|---|---|
| subitem not at all important | ○ | ○ | ○ | ● | ○ | essential |

Clear selection

Does your paper address subitem 14a-i?

Copy and paste relevant sections from the manuscript (include quotes in quotation marks "like this" to indicate direct quotes from your manuscript), or elaborate on this item by providing additional information not in the ms, or briefly explain why the item is not applicable/relevant for your study

"An application developer at the Pennsylvania Department of Health developed and implemented the WIC System as an external cloud system, as opposed to integrated system, as state-wide plans were underway to replace the QuickWIC system."

14b) Why the trial ended or was stopped (early)

Does your paper address CONSORT subitem 14b? *

Copy and paste relevant sections from the manuscript (include quotes in quotation marks "like this" to indicate direct quotes from your manuscript), or elaborate on this item by providing additional information not in the ms, or briefly explain why the item is not applicable/relevant for your study

Not applicable - the trial stopped once the predetermined sample size was obtained, but this is not the focus of this paper.

15) A table showing baseline demographic and clinical characteristics for each group

NPT: When applicable, a description of care providers (case volume, qualification, expertise, etc.) and centers (volume) in each group

Does your paper address CONSORT subitem 15? *

Copy and paste relevant sections from the manuscript (include quotes in quotation marks "like this" to indicate direct quotes from your manuscript), or elaborate on this item by providing additional information not in the ms, or briefly explain why the item is not applicable/relevant for your study

"As shown in Table 2, 49.6% of infants were male and 67.7% were white. Mothers were 27.7±5.7 years old at the time of infant delivery and 26% were primiparous. Infants were enrolled at 0.34±0.43 months. Most mothers were white, single, had an income less than $50,000, and had at least a high school diploma. "

15-i) Report demographics associated with digital divide issues

In ehealth trials it is particularly important to report demographics associated with digital divide issues, such as age, education, gender, social-economic status, computer/Internet/ehealth literacy of the participants, if known.

| | 1 | 2 | 3 | 4 | 5 | |
|---|---|---|---|---|---|---|
| subitem not at all important | ○ | ○ | ○ | ◉ | ○ | essential |

Clear selection

Does your paper address subitem 15-i? *

Copy and paste relevant sections from the manuscript (include quotes in quotation marks "like this" to indicate direct quotes from your manuscript), or elaborate on this item by providing additional information not in the ms, or briefly explain why the item is not applicable/relevant for your study

"As shown in Table 2, 49.6% of infants were male and 67.7% were white. Mothers were 27.7±5.7 years old at the time of infant delivery and 26% were primiparous. Infants were enrolled at 0.34±0.43 months. Most mothers were white, single, had an income less than $50,000, and had at least a high school diploma. " "However, electronic solutions to collect patient-reported information need to account for technology availability at both the patient- and clinic-levels to not exacerbate the digital divide seen between lower and higher income populations[26]."

## 16) For each group, number of participants (denominator) included in each analysis and whether the analysis was by original assigned groups

16-i) Report multiple “denominators” and provide definitions

Report multiple “denominators” and provide definitions: Report N’s (and effect sizes) “across a range of study participation [and use] thresholds” [1], e.g., N exposed, N consented, N used more than x times, N used more than y weeks, N participants “used” the intervention/comparator at specific pre-defined time points of interest (in absolute and relative numbers per group). Always clearly define “use” of the intervention.

| | 1 | 2 | 3 | 4 | 5 | |
|---|---|---|---|---|---|---|
| subitem not at all important | ○ | ○ | ○ | ○ | ● | essential |

Clear selection

### Does your paper address subitem 16-i? *

Copy and paste relevant sections from the manuscript (include quotes in quotation marks "like this" to indicate direct quotes from your manuscript), or elaborate on this item by providing additional information not in the ms, or briefly explain why the item is not applicable/relevant for your study

"The 131 intervention mother-infant dyads" - sample size was the same for each analysis in the study.

### 16-ii) Primary analysis should be intent-to-treat

Primary analysis should be intent-to-treat, secondary analyses could include comparing only "users", with the appropriate caveats that this is no longer a randomized sample (see 18-i).

| | 1 | 2 | 3 | 4 | 5 | |
|---|---|---|---|---|---|---|
| subitem not at all important | ○ | ○ | ○ | ◉ | ○ | essential |

Clear selection

### Does your paper address subitem 16-ii?

Copy and paste relevant sections from the manuscript (include quotes in quotation marks "like this" to indicate direct quotes from your manuscript), or elaborate on this item by providing additional information not in the ms, or briefly explain why the item is not applicable/relevant for your study

Not applicable - the focus of this manuscript is to use descriptive statistics to describe the usability and feasibility of an intervention in the intervention arm only.

### 17a) For each primary and secondary outcome, results for each group, and the estimated effect size and its precision (such as 95% confidence interval)

Does your paper address CONSORT subitem 17a? *

Copy and paste relevant sections from the manuscript (include quotes in quotation marks "like this" to indicate direct quotes from your manuscript), or elaborate on this item by providing additional information not in the ms, or briefly explain why the item is not applicable/relevant for your study

Not applicable - the study does not include any analyses that would call for effect sizes to be produced.

17a-i) Presentation of process outcomes such as metrics of use and intensity of use

In addition to primary/secondary (clinical) outcomes, the presentation of process outcomes such as metrics of use and intensity of use (dose, exposure) and their operational definitions is critical. This does not only refer to metrics of attrition (13-b) (often a binary variable), but also to more continuous exposure metrics such as "average session length". These must be accompanied by a technical description how a metric like a "session" is defined (e.g., timeout after idle time) [1] (report under item 6a).

| | 1 | 2 | 3 | 4 | 5 | |
|---|---|---|---|---|---|---|
| subitem not at all important | ○ | ○ | ○ | ○ | ● | essential |

Clear selection

Does your paper address subitem 17a-i?

Copy and paste relevant sections from the manuscript (include quotes in quotation marks "like this" to indicate direct quotes from your manuscript), or elaborate on this item by providing additional information not in the ms, or briefly explain why the item is not applicable/relevant for your study

"Data Sharing is the electronic and systematic push of study-related data through the electronic data warehouse (distinct from EHR and PCP System) managed by the research team in the clinical setting. Data Sharing does not require the PCP or WIC Nutritionist in the receiving setting to interact with the data of interest (Table 1).
A Care Coordination Opportunity is nested within Data Sharing and is an opportunity for a PCP or WIC Nutritionist to view data for the most recent visit from the sending setting. Due to the secure and study staff-dependent data sharing processes, the receiving provider was able to view the shared data within 2 business days if sent by PCP and 1 business day if sent by WIC. Eligible WCVs occurred after the dyad was enrolled into the study and occurred up to 6 months of infant's age at which time the clinical study team turned off the PCP System. Eligible WIC visits occurred after the dyad was enrolled in the study but before disabling the PCP System.
Care Coordination is nested within Care Coordination Opportunity and requires a PCP or WIC Nutritionist in the receiving setting to be able to access the sent data AND document provided RP care. The study team could not directly verify that the receiving setting interacted with the sent data as accessing or viewing the data was not captured with a discrete data point; thus, the documentation of RP care serves as proxy evidence that the receiving provider used the shared data to coordinate care. Preventive RP care included educational messages that addressed parent learning needs as self-assessed by parents using the EHL tool. Preventive RP care was documented by PCPs and WIC Nutritionists by selecting RP topics and/or writing a free-text comment."

17b) For binary outcomes, presentation of both absolute and relative effect sizes is recommended

Does your paper address CONSORT subitem 17b? *

Copy and paste relevant sections from the manuscript (include quotes in quotation marks "like this" to indicate direct quotes from your manuscript), or elaborate on this item by providing additional information not in the ms, or briefly explain why the item is not applicable/relevant for your study

Not applicable - the study does not include any analyses that would call for effect sizes to be produced.

## 18) Results of any other analyses performed, including subgroup analyses and adjusted analyses, distinguishing pre-specified from exploratory

Does your paper address CONSORT subitem 18? *

Copy and paste relevant sections from the manuscript (include quotes in quotation marks "like this" to indicate direct quotes from your manuscript), or elaborate on this item by providing additional information not in the ms, or briefly explain why the item is not applicable/relevant for your study

Not applicable - the study does not include subgroup or adjusted analyses

18-i) Subgroup analysis of comparing only users

A subgroup analysis of comparing only users is not uncommon in ehealth trials, but if done, it must be stressed that this is a self-selected sample and no longer an unbiased sample from a randomized trial (see 16-iii).

| | 1 | 2 | 3 | 4 | 5 | |
|---|---|---|---|---|---|---|
| subitem not at all important | ○ | ○ | ○ | ◉ | ○ | essential |

Clear selection

Does your paper address subitem 18-i?

Copy and paste relevant sections from the manuscript (include quotes in quotation marks "like this" to indicate direct quotes from your manuscript), or elaborate on this item by providing additional information not in the ms, or briefly explain why the item is not applicable/relevant for your study

Not applicable - the study does not include subgroup analyses

19) All important harms or unintended effects in each group

(for specific guidance see CONSORT for harms)

Does your paper address CONSORT subitem 19? *

Copy and paste relevant sections from the manuscript (include quotes in quotation marks "like this" to indicate direct quotes from your manuscript), or elaborate on this item by providing additional information not in the ms, or briefly explain why the item is not applicable/relevant for your study

Not applicable - the focus paper is to describe the feasibility and usability of the intervention components not the effect of the intervention on participants

19-i) Include privacy breaches, technical problems

Include privacy breaches, technical problems. This does not only include physical “harm” to participants, but also incidents such as perceived or real privacy breaches [1], technical problems, and other unexpected/unintended incidents. “Unintended effects” also includes unintended positive effects [2].

| | 1 | 2 | 3 | 4 | 5 | |
|---|---|---|---|---|---|---|
| subitem not at all important | ○ | ○ | ● | ○ | ○ | essential |

Clear selection

Does your paper address subitem 19-i?

Copy and paste relevant sections from the manuscript (include quotes in quotation marks "like this" to indicate direct quotes from your manuscript), or elaborate on this item by providing additional information not in the ms, or briefly explain why the item is not applicable/relevant for your study

Not applicable - this was not the focus or was captured in the data used for this manuscript

19-ii) Include qualitative feedback from participants or observations from staff/researchers

Include qualitative feedback from participants or observations from staff/researchers, if available, on strengths and shortcomings of the application, especially if they point to unintended/unexpected effects or uses. This includes (if available) reasons for why people did or did not use the application as intended by the developers.

| | 1 | 2 | 3 | 4 | 5 | |
|---|---|---|---|---|---|---|
| subitem not at all important | ○ | ○ | ◉ | ○ | ○ | essential |

Clear selection

Does your paper address subitem 19-ii?

Copy and paste relevant sections from the manuscript (include quotes in quotation marks "like this" to indicate direct quotes from your manuscript), or elaborate on this item by providing additional information not in the ms, or briefly explain why the item is not applicable/relevant for your study

Not applicable - results of qualitative formative phase for this project has already been published.

## DISCUSSION

## 22) Interpretation consistent with results, balancing benefits and harms, and considering other relevant evidence

NPT: In addition, take into account the choice of the comparator, lack of or partial blinding, and unequal expertise of care providers or centers in each group

### 22-i) Restate study questions and summarize the answers suggested by the data, starting with primary outcomes and process outcomes (use)

Restate study questions and summarize the answers suggested by the data, starting with primary outcomes and process outcomes (use).

| | 1 | 2 | 3 | 4 | 5 | |
|---|---|---|---|---|---|---|
| subitem not at all important | ○ | ○ | ● | ○ | ○ | essential |

Clear selection

### Does your paper address subitem 22-i? *

Copy and paste relevant sections from the manuscript (include quotes in quotation marks "like this" to indicate direct quotes from your manuscript), or elaborate on this item by providing additional information not in the ms, or briefly explain why the item is not applicable/relevant for your study

"This study suggests that advanced HIT strategies are a potential solution to engage parents in reporting RP practices, integrate data into the infant's electronic patient management systems to inform and facilitate documentation of provided RP care, and share data between PCPs and WIC Nutritionists serving socioeconomically-disadvantaged parents and infants from an area with known shortages in healthcare services. Even though bi-directional data sharing was feasible, care coordination occurred less frequently. Typically, these advanced HIT strategies are commonly found and siloed in larger health systems with standardized EHRs, a resource supported by policy and practice [11–13]; however, the current study applied advanced HIT strategies to bi-directionally share data across local community and clinical settings, an important step in coordinating patient-centered preventive care. Implemented strategies used to collect, integrate, and share data were adopted by patients, PCPs, and WIC Nutritionists suggesting usability, but variable utilization of certain components inhibited care coordination."

22-ii) Highlight unanswered new questions, suggest future research

Highlight unanswered new questions, suggest future research.

| | 1 | 2 | 3 | 4 | 5 | |
|---|---|---|---|---|---|---|
| subitem not at all important | ○ | ○ | ◉ | ○ | ○ | essential |

Clear selection

Does your paper address subitem 22-ii?

Copy and paste relevant sections from the manuscript (include quotes in quotation marks "like this" to indicate direct quotes from your manuscript), or elaborate on this item by providing additional information not in the ms, or briefly explain why the item is not applicable/relevant for your study

"Replication and dissemination of a cross-sector model may be facilitated by clinical partners practicing in a standardized EHR environment with these findings providing foundational lessons and recommendations for future iterations." "However, passive HIT strategies to collect and extract data can feasibly and effectively facilitate data sharing from a clinical to a non-clinical setting; thus, future iterations of this model should utilize passive, automatic processes in both settings." "Even though the data sharing process between PCPs and WIC was completed in 1 to 2 business days, future applications should aim to shorten this delay and automate the data transfer in real-time. Addressing these limitations would accommodate participants who attend multiple visits within the same day or on consecutive days as well as reduce reliance on human resources thus increasing the generalizability of the model to real-world setting with limited resources. " "Adoption of interventional EHR components may be improved through randomization at the PCP-level, longer exposure times to innovations, additional training and booster sessions, feedback on performance, and enhanced organizational factors, such as administrative and operational support[24,25]." "However, electronic solutions to collect patient-reported information need to account for technology availability at both the patient- and clinic-levels to not exacerbate the digital divide seen between lower and higher income populations[26]."

## 20) Trial limitations, addressing sources of potential bias, imprecision, and, if relevant, multiplicity of analyses

### 20-i) Typical limitations in ehealth trials

Typical limitations in ehealth trials: Participants in ehealth trials are rarely blinded. Ehealth trials often look at a multiplicity of outcomes, increasing risk for a Type I error. Discuss biases due to non-use of the intervention/usability issues, biases through informed consent procedures, unexpected events.

| | 1 | 2 | 3 | 4 | 5 | |
|---|---|---|---|---|---|---|
| subitem not at all important | ○ | ○ | ○ | ○ | ◉ | essential |

Clear selection

Does your paper address subitem 20-i? *

Copy and paste relevant sections from the manuscript (include quotes in quotation marks "like this" to indicate direct quotes from your manuscript), or elaborate on this item by providing additional information not in the ms, or briefly explain why the item is not applicable/relevant for your study

"While a strength of the pragmatic study is observation of real-life patterns of care, a limitation to testing care coordination was that few participants completed the expected 4 WCVs or 2 WIC visits in a six-month observation period. Visit attendance, timing, and the sequence of visit types was not determined or controlled by the study team. Socioeconomically-disadvantaged parents and infants experience many barriers to attending WIC appointments, including lack of transportation or child care, conflicting activities, negative feelings about nutrition education, and frequent relocation[30]. Research in the prenatal period suggests that providing low-income additional supports, such as case managers or outreach/community healthcare workers[31], may enhance efforts to coordinate care between clinical and community settings by promoting timely visit attendance. Although WEE Baby Care was informed by a formative qualitative phase that captured the perceptions of stakeholders, including PCPs, WIC Nutritionists, and parents[4], perspectives of these stakeholders on the implementation of the intervention components were not captured which limited our understanding of how PCPs and Nutritionists integrated the intervention into their workflow. Additional training booster sessions and a longer intervention period may have improved adoption of the PCP and WIC Systems through reminders and increased exposure. Further, the limitations of the WIC System and iterations of this model may be optimized as WIC has implemented an updated, nationally standardized participant management system since the completion of this study; thus, providing an opportunity to improve the technology as well as potential for evaluation and spread. Recruiting dyads after the first WCV likely reduced the number of potential opportunities for data sharing and care coordination. Lastly, sample of participating low-income mothers were primarily White, all were English-speaking, and most had a high school diploma or higher, which were representative of the Geisinger region but generalizability to the more diverse national WIC and low-income populations with lower educational attainment may be limited[32,33]."

## 21) Generalisability (external validity, applicability) of the trial findings

NPT: External validity of the trial findings according to the intervention, comparators, patients, and care providers or centers involved in the trial

### 21-i) Generalizability to other populations

Generalizability to other populations: In particular, discuss generalizability to a general Internet population, outside of a RCT setting, and general patient population, including applicability of the study results for other organizations

| | 1 | 2 | 3 | 4 | 5 | |
|---|---|---|---|---|---|---|
| subitem not at all important | ○ | ○ | ○ | ● | ○ | essential |

Clear selection

### Does your paper address subitem 21-i?

Copy and paste relevant sections from the manuscript (include quotes in quotation marks "like this" to indicate direct quotes from your manuscript), or elaborate on this item by providing additional information not in the ms, or briefly explain why the item is not applicable/relevant for your study

"Lastly, sample of participating low-income mothers were primarily White, all were English-speaking, and most had a high school diploma or higher, which were representative of the Geisinger region but generalizability to the more diverse national WIC and low-income populations with lower educational attainment may be limited[32,33]."

### 21-ii) Discuss if there were elements in the RCT that would be different in a routine application setting

Discuss if there were elements in the RCT that would be different in a routine application setting (e.g., prompts/reminders, more human involvement, training sessions or other co-interventions) and what impact the omission of these elements could have on use, adoption, or outcomes if the intervention is applied outside of a RCT setting.

| | 1 | 2 | 3 | 4 | 5 | |
|---|---|---|---|---|---|---|
| subitem not at all important | ○ | ○ | ● | ○ | ○ | essential |

Clear selection

Does your paper address subitem 21-ii?

Copy and paste relevant sections from the manuscript (include quotes in quotation marks "like this" to indicate direct quotes from your manuscript), or elaborate on this item by providing additional information not in the ms, or briefly explain why the item is not applicable/relevant for your study

"Adoption of interventional EHR components may be improved through randomization at the PCP-level, longer exposure times to innovations, additional training and booster sessions, feedback on performance, and enhanced organizational factors, such as administrative and operational support[24,25]. "
"Further innovations in HIT strategies are needed to more efficiently and effectively share limited patient data sets with aligned community and public health agencies to address limitations related to workflow, human resources, and timeliness. "

## OTHER INFORMATION

### 23) Registration number and name of trial registry

Does your paper address CONSORT subitem 23? *

Copy and paste relevant sections from the manuscript (include quotes in quotation marks "like this" to indicate direct quotes from your manuscript), or elaborate on this item by providing additional information not in the ms, or briefly explain why the item is not applicable/relevant for your study

"https://clinicaltrials.gov/ct2/show/NCT03482908. Registered March 29, 2018"

### 24) Where the full trial protocol can be accessed, if available

Does your paper address CONSORT subitem 24? *

Cite a Multimedia Appendix, other reference, or copy and paste relevant sections from the manuscript (include quotes in quotation marks "like this" to indicate direct quotes from your manuscript), or elaborate on this item by providing additional information not in the ms, or briefly explain why the item is not applicable/relevant for your study

"Savage JS, Kling SMR, Cook A, Hess L, Lutcher S, Marini M, et al. A patient-centered, coordinated care approach delivered by community and pediatric primary care providers to promote responsive parenting: Pragmatic randomized clinical trial rationale and protocol. BMC Pediatr. 2018;18(1). PMID: 30180831"

## 25) Sources of funding and other support (such as supply of drugs), role of funders

Does your paper address CONSORT subitem 25? *

Copy and paste relevant sections from the manuscript (include quotes in quotation marks "like this" to indicate direct quotes from your manuscript), or elaborate on this item by providing additional information not in the ms, or briefly explain why the item is not applicable/relevant for your study

"This project is supported by the Health Resources and Services Administration (HRSA) of the U.S. Department of Health and Human Services (HHS) under grant number R40MC28317, Maternal and Child Health Field-initiated Innovative Research Studies Program. Under the umbrella of becoming a learning healthcare system, Geisinger has funded internal quality improvement grants to provide iPads and kiosks to clinics."

## X27) Conflicts of Interest (not a CONSORT item)

X27-i) State the relation of the study team towards the system being evaluated

In addition to the usual declaration of interests (financial or otherwise), also state the relation of the study team towards the system being evaluated, i.e., state if the authors/evaluators are distinct from or identical with the developers/sponsors of the intervention.

| | 1 | 2 | 3 | 4 | 5 | |
|---|---|---|---|---|---|---|
| subitem not at all important | ○ | ○ | ○ | ○ | ◉ | essential |

Clear selection

Does your paper address subitem X27-i?

Copy and paste relevant sections from the manuscript (include quotes in quotation marks "like this" to indicate direct quotes from your manuscript), or elaborate on this item by providing additional information not in the ms, or briefly explain why the item is not applicable/relevant for your study

"The authors declare that they have no conflicts of interest."

## About the CONSORT EHEALTH checklist

As a result of using this checklist, did you make changes in your manuscript? *

- ○ yes, major changes
- ○ yes, minor changes
- ◉ no

What were the most important changes you made as a result of using this checklist?

Not applicable - Did not make major changes as 1) this was a secondary analysis of an RCT and 2) we had used the CONSORT checklist throughout the study.

How much time did you spend on going through the checklist INCLUDING making changes in your manuscript *

2 hours

As a result of using this checklist, do you think your manuscript has improved? *

- ○ yes
- ◉ no
- ○ Other:

Would you like to become involved in the CONSORT EHEALTH group?

This would involve for example becoming involved in participating in a workshop and writing an "Explanation and Elaboration" document

- ○ yes
- ◉ no
- ○ Other:

Clear selection

Any other comments or questions on CONSORT EHEALTH

This was too long and difficult to navigate and edit. The pdf versions of checklists used by other journals are much easier to use and can be edited and changed by multiple co-authors.

## STOP - Save this form as PDF before you click submit

To generate a record that you filled in this form, we recommend to generate a PDF of this page (on a Mac, simply select "print" and then select "print as PDF") before you submit it.

When you submit your (revised) paper to JMIR, please upload the PDF as supplementary file.

Don't worry if some text in the textboxes is cut off, as we still have the complete information in our database. Thank you!

## Final step: Click submit !

Click submit so we have your answers in our database!

Submit

Never submit passwords through Google Forms.

This content is neither created nor endorsed by Google. Report Abuse - Terms of Service - Privacy Policy

Google Forms
